# Supplementary figures and images for: Health system actors’ perspectives of prescribing practices in public health facilities in Eswatini: A Qualitative Study
Source: PLoS One. 2020 Jul 9;15(7):e0235513. doi: 10.1371/journal.pone.0235513 (PMC7347100; doi:10.1371/journal.pone.0235513)

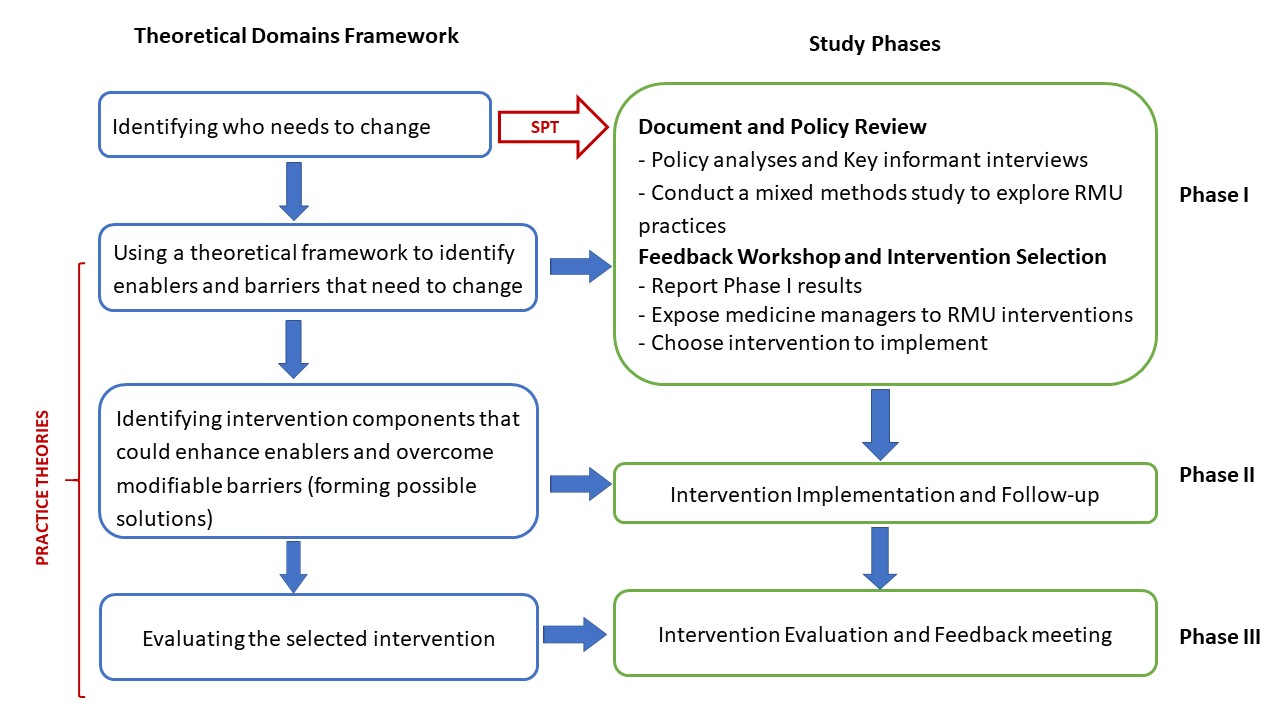


Fig 1. Study Theories and Procedures Key: SPT – Social Practice Theory

Supplement: S1 Fig — (DOCX) [file pone.0235513.s001.docx]
