# Supplementary material for: Health system actors’ perspectives of prescribing practices in public health facilities in Eswatini: A Qualitative Study
Source: PLoS One. 2020 Jul 9;15(7):e0235513. doi: 10.1371/journal.pone.0235513 (PMC7347100; doi:10.1371/journal.pone.0235513)
Supplement: S1 Table — (DOCX) [file pone.0235513.s002.docx]

Table 1. Factors affecting prescribing behaviours and how they link to the SPT

| **Type of Factor and SPT Element** | **Factors** |
| --- | --- |
| Provider (Competence) | Practitioner’s experience and level of education, inadequate training of healthcare providers, visits by pharmaceutical sales representative [6–8], physician’s age and gender [9, 10], prescribers’ perception that the patient wants certain medicines and fear that not giving antibiotics will lead to patients having medical complications [11, 12], fear that prescribers will lose patients if they do not give in to their demands [13]. |
| Patient-provider interaction (shared meaning) | Patient expectations and demands [13]. |
| Health system context (Material) | Health systems factors such as lack of continuous professional development, lack of structures that provide updated and evidence-based information on medicines in current use, patient load, and influences of pharmaceutical representatives [6–8],  socio-economic characteristics of the environment in which practice is conducted, health demand [9], availability of materials to work with [14, 15], and availability of funds to procure medicines [16]. |
