# Supplementary material for: Health system actors’ perspectives of prescribing practices in public health facilities in Eswatini: A Qualitative Study
Source: PLoS One. 2020 Jul 9;15(7):e0235513. doi: 10.1371/journal.pone.0235513 (PMC7347100; doi:10.1371/journal.pone.0235513)
Supplement: S2 Table — (DOCX) [file pone.0235513.s003.docx]

Table 2. Study participants’ characteristics

| Characteristics | Cadre (Number and Region) |
| --- | --- |
| Key Informants (N=7)  Ministry of Health (National level of care)  Implementing Partner  Ministry of Health (Central level of care)  Ministry of Health (Regional level of care) | Pharmacist (1: Hhohho region)  Pharmacist (1: Hhohho region)  Pharmacist (1)  Nursing Matron (4: 1 from each region) |
| Frontline Managers (N=32)  Ministry of Health (Secondary level of care)  Ministry of Health (Primary level of care) | Medical Officer (1: Lubombo region)  Pharmacist (2: 1 from Hhohho and 1 from Shiselweni regions)  Pharmacy Technicians (6: 4 from health centres [2 from Hhohho; 2 from Shiselweni] and 2 from hospitals [1 from Shiselweni and 1 from Manzini])  Nurse (23: 6 from Manzini; 5 from Shiselweni; 5 from Lubombo; 7 from Hhohho)* |

*one clinic from the Hhohho region was wrongly coded as a facility in the Lubombo region by the central medical stores - it was analyzed under the Hhohho region. One clinic in the Shiselweni region was closed during data collection.
