# Supplementary material for: Health system actors’ perspectives of prescribing practices in public health facilities in Eswatini: A Qualitative Study
Source: PLoS One. 2020 Jul 9;15(7):e0235513. doi: 10.1371/journal.pone.0235513 (PMC7347100; doi:10.1371/journal.pone.0235513)
Supplement: S2 File — (DOCX) [file pone.0235513.s005.docx]

**Appendix 11: Semi-structured Interview Guide for National Department of Health, Implementing partner, and Central Medical Stores Medicine Managers**

1. What in your opinion is rational medicine use (RMU)?
2. How do you think RMU applies to consumers/patients?
3. What is the interaction between the NDoH/ CMS and prescribers in facilities with regard to RMU?
4. Are you aware of any interventions that the country has in place to promote RMU?
5. Please describe any interventions that the country has in place to promote RMU:

*Prompts:*

- Standard treatment guidelines?
- Who has been involved?
- How satisfactory was the intervention?
- Could you please describe what happened?
- Pharmaceutics and Therapeutics Committees?
- Who has been involved?
- How satisfactory was the intervention?
- Could you please describe what happened?
- Training?
- Who has been involved?
- How satisfactory was the intervention?
- Could you please describe what happened?

1. What do you think are the benefits of using medicines rationally?
