# Supplementary material for: Health system actors’ perspectives of prescribing practices in public health facilities in Eswatini: A Qualitative Study
Source: PLoS One. 2020 Jul 9;15(7):e0235513. doi: 10.1371/journal.pone.0235513 (PMC7347100; doi:10.1371/journal.pone.0235513)
Supplement: S3 File — (DOCX) [file pone.0235513.s006.docx]

**Appendix 6: Semi-structured Interview Guide for Facility Medicine Managers**

1. What in your opinion is rational medicine use (RMU)?
2. How do you think RMU applies to consumers/patients?
3. Are there any challenges regarding the rational use of medicines in your facility?
4. Are you aware of any interventions in place in your facility to promote RMU?
5. Describe any interventions that your facility has been involved in promoting RMU:

*Prompts:*

- Standard treatment guidelines?
- Who has been involved?
- How satisfactory was the intervention?
- Could you please describe what happened?
- Pharmaceutics and Therapeutics Committees?
- Who has been involved?
- How satisfactory was the intervention?
- Could you please describe what happened?
- Training?
- Who has been involved?
- How satisfactory was the intervention?
- Could you please describe what happened?

1. How do you think your facility would benefit if medicines were used rationally?
