# Supplementary material for: Health system actors’ perspectives of prescribing practices in public health facilities in Eswatini: A Qualitative Study
Source: PLoS One. 2020 Jul 9;15(7):e0235513. doi: 10.1371/journal.pone.0235513 (PMC7347100; doi:10.1371/journal.pone.0235513)
Supplement: S4 File — (DOC) [file pone.0235513.s007.doc]

# Project: Baseline Survey Key Informants

Report created by NONDUMISO on 2/2/2018

**Quotation Report**

All (60) quotations

**1:1 It is ensuring that the right medicines are prescribed for the right c…… (400:501) - D 1: KI_BM_CMS**

It is ensuring that the right medicines are prescribed for the right condition and the right patient.

**1:2 Yes we are, though not 100%. For instance, with antibiotics there are…… (605:877) - D 1: KI_BM_CMS**

Yes we are, though not 100%. For instance, with antibiotics there are no sensitivity tests done before prescribing. Therefore we are not sure if the prescribed antibiotic is effective in treating the patient or whether the patient is resistant to the prescribed antibiotic.

**1:3 I can say that there is appropriate use of medicines at hospital and h…… (885:1503) - D 1: KI_BM_CMS**

I can say that there is appropriate use of medicines at hospital and health centre levels since pharmacy personnel such as Pharmacists and Pharmacy Technicians handle medicines. I am not sure if rational medicines use is practiced at clinic level because in some facilities you find that non-medical staff such as orderlies are handling the medicines. We cannot blame the clinics though because you find that they are under-staffed; the same nurse who prescribes must leave their station and go dispense medicines from the pharmacy. This is not always possible due to the large numbers of patients that have to be seen.

**1:4 We have the STG/EML that is being used by all facilities. In bigger fa…… (1631:1837) - D 1: KI_BM_CMS**

We have the STG/EML that is being used by all facilities. In bigger facilities (hospitals and health centres) we have Pharmaceutics and Therapeutics Committees (PTCs) though most of them are not functional.

**1:5 First of all, the CMS or NDoH are not the ones who add new medicines t…… (2026:2529) - D 1: KI_BM_CMS**

First of all, the CMS or NDoH are not the ones who add new medicines to the list. Doctors from the facilities motivate for the medicine to be added and they include evidence on why they feel the medicine should be added. The motivations come from facilities to the National Essential Medicines List Committee (NEMLC), which then approves the motivation for addition of that item. Once that item has been approved, it then undergoes the tender process before it can be procured and available for ordering.

**1:6 In bigger facilities (hospitals and health centres) we have Pharmaceut…… (1689:1837) - D 1: KI_BM_CMS**

In bigger facilities (hospitals and health centres) we have Pharmaceutics and Therapeutics Committees (PTCs) though most of them are not functional.

**1:7 There are pharmacists in the facilities so they help to review the mot…… (2696:2822) - D 1: KI_BM_CMS**

There are pharmacists in the facilities so they help to review the motivation documents before they are submitted to the NEMLC.

**1:8 They are handled at the regional level through the Regional Health Man…… (2938:3181) - D 1: KI_BM_CMS**

They are handled at the regional level through the Regional Health Management Team (RHMT). At the moment only two regions have Regional Pharmacists who handle the motivations. In the other two regions, Clinic Supervisors handle the motivations.

**1:9 No, I don’t think so. I think there are challenges with stock ordering…… (3296:3504) - D 1: KI_BM_CMS**

No, I don’t think so. I think there are challenges with stock ordering from the facilities. You find that they run out because they haven’t ordered the right quantities due to poor stock management practices.

**1:10 Yes, we conduct a lot of trainings for the regions. We do this by regi…… (3746:4351) - D 1: KI_BM_CMS**

Yes, we conduct a lot of trainings for the regions. We do this by region and the trainings normally last for a week. We invite facility representatives to a central place. We make an effort to ensure that the facilities continue functioning while we conduct trainings by not inviting all facilities at the same time but break them into groups. Pharmacists from the Ministry of Health, with support from the partners, conduct these trainings. However, there are a lot of rotations of nurses within and between facilities hence one never knows whether all facilities have nurses who have been trained on RMU.

**1:11 Yes, we conduct a lot of trainings for the regions. We do this by regi…… (3746:4353) - D 1: KI_BM_CMS**

Yes, we conduct a lot of trainings for the regions. We do this by region and the trainings normally last for a week. We invite facility representatives to a central place. We make an effort to ensure that the facilities continue functioning while we conduct trainings by not inviting all facilities at the same time but break them into groups. Pharmacists from the Ministry of Health, with support from the partners, conduct these trainings. However, there are a lot of rotations of nurses within and between facilities hence one never knows whether all facilities have nurses who have been trained on RMU.

**1:12 There has to be proper pharmaceutical personnel deployed to all facili…… (4463:5156) - D 1: KI_BM_CMS**

There has to be proper pharmaceutical personnel deployed to all facilities. The clinics need to have pharmacy assistants at the minimum. We are aware that Southern African Nazarene University produces this cadre of personnel, though most of them tend to then upgrade to Pharmacy Technicians once they qualify as assistants. I am not sure if our government can afford to have Pharmacy Technicians in all clinics. This intervention might not be financially sustainable. We also need to have functional PTCs in the bigger facilities. What has been a big challenge in the functioning of PTCs in facilities is that doctors do not want to be involved in the PTC. It ends up being a “pharmacy thing”.

**1:13 However, the challenges of not having pharmacy personnel and having no…… (5160:5529) - D 1: KI_BM_CMS**

However, the challenges of not having pharmacy personnel and having no-functional PTCs have been looked at by a task team that was commissioned by the Prime Minister in May this year. The team visited facilities around the country between May and July, and came up with a report on the findings. The report also had recommendations, which have been approved by Cabinet.

**1:14 The recommendations state that at least165 pharmacy assistants need to…… (5533:6027) - D 1: KI_BM_CMS**

The recommendations state that at least165 pharmacy assistants need to be deployed to facilities. Also, the Directorate needs to ensure that PTCs are active and functional in bigger facilities. The Senior Medical Officer in each facility must ensure that PTC meetings take place. As evidence that these meetings are happening, minutes from these meetings (with all issues discussed) must be documented and sent to the office of the Director of Health services and the Chief Pharmacist copied in.

**2:1 We have put in place some contingency measures to try and guide or to…… (431:857) - D 2: KI_FF_CMS**

We have put in place some contingency measures to try and guide or to try and coerce people to use medicines rationally by putting in place standard treatment guidelines. But when we follow with our facilities we have realised that it is not used as such because some of them when you visit facilities they have to look for the STG within their cabinets and stuff yet you'd expect it on the desk where they are working with it.

**2:2 and then when we ask them why they are not using them that much, some…… (892:1100) - D 2: KI_FF_CMS**

and then when we ask them why they are not using them that much, some say they do refer to it from time to time but they say it's not user friendly, it's difficult to carry it around it's too bulky a document

**2:3 At tertiary level they say our STG is skewed a lot towards primary hea…… (1165:1670) - D 2: KI_FF_CMS**

At tertiary level they say our STG is skewed a lot towards primary health care and doesn’t provide for their guidance. and of course it is a little bit true there is some truth to it because you know at tertiary level we can do with a little bit of a revision and we do both tertiary level and primary health care level, because at primary health care level we know that we do not have medical practitioners so we thought those are the ones that needed the guidance the most that's why we started with them

**2:4 But as we review the document we are going to do a document for both l…… (1673:1966) - D 2: KI_FF_CMS**

But as we review the document we are going to do a document for both levels of care and then we are thinking of going with technology to improve the user-friendliness if we can maybe have an app for it so that people can access it from their mobile phones maybe it will be so much easier to use

**2:5 Because people think that […pause…] I don't know it has not been prove…… (1969:2343) - D 2: KI_FF_CMS**

Because people think that […pause…] I don't know it has not been proven so I want to believe it's a perception of the health care workers because they tend to think that if they flip through their STG the patient will think they do not know, each time they are treating them they have to be checking but maybe with a mobile app on their smart phone the fears will be allayed.

**2:6 But yeah to try and improve that we have put in place some Pharmacy an…… (2408:3890) - D 2: KI_FF_CMS**

But yeah to try and improve that we have put in place some Pharmacy and Therapeutics Committees especially when it comes to chronic medications - that is where we have observed the worst case the most because you find that the prescriber before even the chronic patient comes in for their diabetes or hypertension, the prescriber has started writing something on their prescription book the first two being a pain killer or even some multivite without assessing the patient and not knowing what is wrong with them. I mean does it mean everyone who leaves the hospital must have a pain killer, does it mean everyone is in pain - so that's why we say we have picked up elements of irrational use which we need to tell people to solve, encourage those PTCs to try and discourage that habit. Let them see their patient and hear them out before you start tabulating a prescription for that patient because you know I mean polypharmacy doesn't help the patient because they tend to accumulate the medicines in their homes and they have mini pharmacies in their home. Evidence of that is that at times when they are given some of these, because they know their core medicines that they need to take, and then the others they will tell you “oh I still have that at home”, so it shows that they are keeping it they're not taking it they are just taking their BP medication because that is their basic medication so they know that the others are just for pain if I may call them that[…Laughs…]

**2:7 So yeah we are trying our level best to discourage that and we also ha…… (3925:4415) - D 2: KI_FF_CMS**

So yeah we are trying our level best to discourage that and we also have these monthly meetings where we hope again we are going to be preaching the gospel because there we normally have the SMOs and administrators of facilities meeting on a monthly basis so that we hammer the need to make their Pharmacy and therapeutics committee functional on the ground and to monitor the rational use of medicines because with the prevailing fiscal situation I doubt we can still afford to be wasting.

**2:8 yes, normally it’s the Minister who calls these meetings for hospitals…… (4607:4960) - D 2: KI_FF_CMS**

yes, normally it’s the Minister who calls these meetings for hospitals and health centres - we still need a way to reach out to the clinics. So we hope with the approval of the posts for Regional pharmacists - that will be the forum because we have them attend these meetings and stress the importance of rational use of medicines during these meetings.

**2:9 and another way to try and address that at clinical level is we have a…… (4996:5253) - D 2: KI_FF_CMS**

and another way to try and address that at clinical level is we have asked for posts for pharmacy assistants at clinic level to be manning the medicines. We haven't received any yet but we are still waiting to hear if there is anything that has come our way.

**2:10 There are plenty actually because just as we’re speaking, we are worki…… (5410:6182) - D 2: KI_FF_CMS**

There are plenty actually because just as we’re speaking, we are working on an antimicrobial resistance strategy, a national one, because you know again we believe that the irrational use of medicines has increased the incidence of resistant strains towards our antimicrobials. We know that there is natural resistance that develops with prolonged use of an antimicrobial but we believe that that accounts for a minimal percentage but most of the resistance we see emerges from the irrational use of our antimicrobials. And of course there is need from a financial perspective because there is wastage of medication when we have to address the real ailments and underlying ailments. But then if we are using them irrationally then the disease burden keeps on piling on us.

**2:11 We haven't done any trainings but we are hoping that in the near futur…… (6346:6737) - D 2: KI_FF_CMS**

We haven't done any trainings but we are hoping that in the near future will be doing them because we are believing that if we can start up with the updated version of the standard treatment guidelines and as we launch them we do trainings along with the launching on rational use of medicines. We are believing it will be the one with an app and teach people how to use it and stress the use

**2:12 We have what we call a national essential medicines committee that loo…… (7021:8188) - D 2: KI_FF_CMS**

We have what we call a national essential medicines committee that looks at the use of medicine and that looks at the need to be reviewing our standard treatment guidelines – that (the committee) pick up motivations from facility level on the addition or deletion of certain medicines in the essential medicines list so that’s the one that links with facilities. So facilities make a proposal to add or delete and then we invite that person to come and give information on the prons and cons of what has to be added and how is it superior over and above what we already have on the ground. especially if it has to be added, not like it's a replacement because now we need a stronger motivation and a stronger recommendation. This then links back to the pharmacist at the respective health facility because they are the ones that bring in the information then we give them the feedback and they take it back to their Pharmacy and Therapeutics Committee because that’s where the information is coming from. The Pharmacy and Therapeutics Committees are the ones that do the motivation and send it up to the committee and then we respond and send it back to the facility.

**2:13 We haven't reached the consumer level but we have this adverse drug re…… (8646:9723) - D 2: KI_FF_CMS**

We haven't reached the consumer level but we have this adverse drug reporting. For now adverse drug reporting is happening at facility level if I may call it that because it’s the health workers reporting to the pharmacovigilance unit about adverse drug reactions that patients are reporting but we feel that there is under-reporting. So we are looking at ways of getting patients to report themselves, as a system where patients can report themselves, because we believe that way we can pick up a lot of reporting. So probably at that stage we can then go out and sensitize patience. Secondly, the sensitization of patients, we hope to reach patients because of the enactment of the Medicines Act as we hope to do a lot of community sensitization on this Act. Once we sensitize them to this Act we could also talk about the rational use of medicines. I know that the Minister proposed that we visit patients’ homes and retrieve the medicines […laughs…] but that was a cumbersome exercise. We haven't shelved it, but we’re still trying to see how feasible it will be to do that.

**2:14 we are thinking of ways of reaching communities for instance we were t…… (10381:10787) - D 2: KI_FF_CMS**

we are thinking of ways of reaching communities for instance we were thinking of discussing with schools because we believe that when you teach the kids they tend to grasp something and they tend to be the ones who then teach their parents because they were taught that thing at school. So if we catch them young they will grow up with these good practices - that is another avenue that we have thought of.

**2:15 yes...of course there is always room for improvement because if we can…… (10957:11786) - D 2: KI_FF_CMS**

yes...of course there is always room for improvement because if we can be talking to people let's say reaching the communities through some radio show because I have noticed and listened to a slot that we have in the morning. When you talk to people you do pick up the interest, but probably that morning slot is not enough because not a lot of people are up at that time. But if we can find a slot somewhere to be telling people on the dangers of using medicines irrationally because I believe that with some people it's due to lack of knowledge of the fact that irrational use of medicines can harm them, they don't know the adverse effects that the medicines can have on them so I believe that there is room for improvement. We tend to focus a lot on the health care workers, but I think there is a bigger gap on the community.

**3:1 I think in the context of what we work with in Swaziland especially in…… (270:1007) - D 3: KI_KM_MSH**

I think in the context of what we work with in Swaziland especially in the public sector it’s really about the person - be it the prescriber or the user using the medicine appropriately or from a prescribers’ point of view it will be uhm someone who is a prescriber prescribing the medicine for the right patient, for the right condition, and in the appropriate dosage. And obviously with the patient, it will be now using the medicine as advised by the prescriber and again looking at the duration of the treatment and also the frequency of the treatment. So it relies on the prescriber following the protocols that are in existence and also the patient following the guidance and advice on how to use the medication that they are given.

**3:2 I would say an organisation or as a person, we have been supporting th…… (1232:2502) - D 3: KI_KM_MSH**

I would say an organisation or as a person, we have been supporting the ministry of health since 2010 in developing the treatment protocols. So we developed a standard treatment guideline for common illnesses in Swaziland and also the essential medicine list. With respect to standard treatment guidelines, there were various disease specific standard treatment guidelines before the one we developed - so there was HIV and IMCI and there was also a TB guideline. But we put together treatment guidelines that would cover everything especially the common illnesses; and the reason why we started that project of developing the standard treatment guidelines - we realised that most of our physicians in the country are trained outside the country. So doctors come from South African training institutes, others from Kenya, Uganda, Zimbabwe and they bring in what they know or how they were taught to prescribe, to treat conditions. So we felt that as part of improving the rational medicine use we need to start by developing a standard treatment guideline and the essential medicine list. So we have been supporting that from the development of the guideline, supporting the implementation of the guideline, and hopefully these guidelines will be due for a review soon.

**3:3 The process - obviously it had participation of all the health profess…… (2663:3849) - D 3: KI_KM_MSH**

The process - obviously it had participation of all the health professional, so we got to get a go ahead from the ministry of health (the principal secretary who is the head of the ministry), to establish a committee or a task team that will oversee the development of the guideline. This task team had representatives from the nursing, doctors, and the pharmacy healthcare cadres. We had people coming in from ehhh, we had members from the private sector that brought in certain expertise like paediatricians, and physicians, internal medicine specialists and the committee had sort of a subcommittee - people that would look at each section; paediatric section, adult sections, emergency medical care. I almost forgot there were academics - so we got people from the universities participating in the process to also bring in that angle because we wanted to make sure that it is not just about having the guidelines at facilities but we wanted to see the next cohort of nurses (which is what we train in the country), being trained on the guideline. So they need to be familiar with the guideline, how diseases are treated in Swaziland, and what medicines are being used in Swaziland.

**3:4 So we had representatives from the academia and we had representatives…… (3851:5367) - D 3: KI_KM_MSH**

So we had representatives from the academia and we had representatives from medical and dental association of doctors in Swaziland as well as the nursing association because our whole aim actually was to make sure that it is owned by the practitioners - it is owned by the people that will be using it and that they see it as if it’s their document. We did not want to use a top-down approach. We wanted to as far as possible bring it from the bottom to up so even if the evidence is, even if the current practice is saying do A, B, C, D in treating the disease we wanted to make sure that, that practice can be well informed by the science of it but also if we are to change that practice we have to convince the leaders at the head of the nursing institution, at health facility and academic institution that this is the latest practice so lets’ move together. So ownership to us was very important and actually the process took very long because we wanted to make sure that everyone was consulted and if you look at the guideline that we developed it’s like two pages, the front two pages is a list of the people who participated. So that even if people say that “ah I don’t even know these guidelines, who came up with them”, we can actually say ah your name is here (laughs) or your facility is involved, or your profession was represented. So to us we felt that the big part more than anything, more than the science of it in informing the guideline and essential medical list, ownership was the most important.

**3:5 So we had representatives from the academia and we had representatives…… (3851:5366) - D 3: KI_KM_MSH**

So we had representatives from the academia and we had representatives from medical and dental association of doctors in Swaziland as well as the nursing association because our whole aim actually was to make sure that it is owned by the practitioners - it is owned by the people that will be using it and that they see it as if it’s their document. We did not want to use a top-down approach. We wanted to as far as possible bring it from the bottom to up so even if the evidence is, even if the current practice is saying do A, B, C, D in treating the disease we wanted to make sure that, that practice can be well informed by the science of it but also if we are to change that practice we have to convince the leaders at the head of the nursing institution, at health facility and academic institution that this is the latest practice so lets’ move together. So ownership to us was very important and actually the process took very long because we wanted to make sure that everyone was consulted and if you look at the guideline that we developed it’s like two pages, the front two pages is a list of the people who participated. So that even if people say that “ah I don’t even know these guidelines, who came up with them”, we can actually say ah your name is here (laughs) or your facility is involved, or your profession was represented. So to us we felt that the big part more than anything, more than the science of it in informing the guideline and essential medical list, ownership was the most important

**3:6 so in launching the guideline and this has been an issue really, since…… (5530:6180) - D 3: KI_KM_MSH**

so in launching the guideline and this has been an issue really, since the guidelines were introduced that how do you train people (laughs) you know, we have the, I mean when we studied at our undergraduate, you were given the British medicine formulary, or the South African medicine formulary no one taught you on a workshop on how to use it. But anyway our approach to the implementation we wanted to do top A, [NN1]we wanted to take extracts of the guidelines for certain diseases areas that are most common, so if we were to say hypertension. We just underlined it A but we deal with hypertension only just as a good reference for those diseases

**3:7 then we also got to do on site training, where during our nurses break…… (6211:8746) - D 3: KI_KM_MSH**

then we also got to do on site training, where during our nurses break we would sit down and talk about whatever we have seen as not being managed appropriately or according to the guideline so that was the approach that we used, umm with the guideline, but there wasn’t a lot of umm prep for training and in the facility we go to, people would say “oh no we have never been trained on this guideline” and I really have never been convinced that that is something you can train people on. We did an orientation to say this is the guideline, these are the chapters, these are the disease programmes that are here, and these conditions you refer to the next level these conditions you manage at facilities, from the essential medical list these are the products that the nurses can prescribe, and these are the products that doctors can prescribe. Just a quick overview but it wasn’t something that we can say people have been trained on it. We introduced it at universities, so we got to work with the universities, to have it as part of their reference books and have it at their faculty, or library so that at least the students have access to the document and can be able to reference it. Umm, its available on line, we have a copy in the government website of Swaziland. We also printed hard copies and gave them CDs so that at least if people have computers, they can just put it on their computer and it stays there because what we have seen on the guideline is that we have ehh, they have become, they just sit there, and people leave, you know, yah. So I think our approach to implementation was really that, but because it was the first treatment guideline we wanted to make sure we get as much feedback as possible, we hear what people say about the guidelines, are they user friendly and are you able to carry them around, are you able to find what you are looking for and we got some good feedback. People say no but this condition is not included in the guideline, this condition the protocol that is in the treatment guideline is not sufficient and we also got feedback like other departments like hospitals because our guidelines are mainly for common conditions so it’s mainly primary health care. So we got feedback that hospitals also want guidelines as well, that they need guidelines. So the reception was good, people were seeing the value of having the guideline but obviously there is still room to improve on the use of the guideline. Because people know out there but they may not be using them as they should.

**3:8 so we took a conscious decision not to focus on the hospital because a…… (8960:11338) - D 3: KI_KM_MSH**

so we took a conscious decision not to focus on the hospital because as I have said in the introduction, we especially the doctors are trained outside the country, so we wanted to make sure we standardise the treatment protocols in the country. But during the development of the guideline we realised that it is a complex process to develop guidelines that will now address the higher-level conditions - sort of tertiary conditions. Hence we decided that at the first stage; let’s focus on the primary health care for the doctors and nurses. Hospitals have really nothing at this stage because that will mean that if there is a gynae in a hospital, the gynae would want a guideline that will stick on what he does or what he knows in the facility umm and when that gynae leaves the next one will come and have another protocol of managing the disease condition so it meant that it will probably take us a little more time to get to a point where these guidelines are developed for a tertiary condition and for specialists to start adhering to them, and that requires also government supervision where government will make sure that no one out there cries or uses a drug that is not in the essential medicine list or manages the condition in a way that is not in the standard treatment guideline because doctors sometimes have that kind of freedom at times to prescribe what they want, demand the drugs that they feel like they want to use umm, uhm so yah in the hospital there isn’t much except for the few guidelines that the paediatrician will develop on how to manage whatever condition that is common in that facility. Then the obscene gynae will develop his own guideline but what happens then is that if the drug is not, if that drug that comes in that guideline is not in the essential medicine list because we try by all means that at least the essential medical list will be all incapacitated, come with all medicines that can be possibly used in Swaziland. So if the drug that they want to introduce is not in the essential medical list, they will then have to write an application and request the inclusion of that drug on the essential medicinal list and then based on the science, the efficiency, the cost effectiveness that product will then be included in the essential medical list and the government will start buying it umm as part of the national protocol.

**3:9 so what normally happens is the facilities are supposed to have pharma…… (11832:14412) - D 3: KI_KM_MSH**

so what normally happens is the facilities are supposed to have pharmacy therapeutic committee so all the hospitals are supposed to have pharmacy therapeutic committee and if there is a specialist; if we make an example of a specialist, if a specialist want to include a certain drug and that drug is not on the essential medicine list, he will have to then come up with a justification. So there is a form on the standard treatment guideline that the paediatrician can complete and ehh then submit that form completed with the science, if there is any literature that supports this drug and then submit it to the pharmacy therapeutic committee which is in their facility, stating that this is the drug I would like to use, I would like to have access to the drug for my patients with this condition, because it is superior to the drug that is already in the essential medical list or its not in the list, there is no equivalent drug on the list. Once the pharmacy therapeutic committee review that and approve it they then submit it through the office of the chief pharmacist to the national essential medicines committee. The national essential medicines committee will then look at the evidence, and the motivation as well, then if it is satisfied that this is sufficient and indeed it will be beneficial because we also don’t want to look at the case of one hospital at the national essential medical list we are looking at the whole country so we have to look at the evidence in the context of the whole country and then if approved by the national essential medicine committee, it will be included in the essential medical list and it becomes part of the national government protocol, and so the national essential medicine list will also indicate the prescriber level, that is the prescriber level for a drug like this needs to be prescribed by a specialist, especially if it needs to have some micro sensitivity as seen in the case of antibiotics that will also be included as well, so that is how it is done. But unfortunately it hasn’t been followed that way because facilities don’t have pharmacist therapeutic committee, they don’t really, they are not active. And at the national essential medicine committee though we may have the people because it’s a committee with academics and also we have pharmacists, doctors. But at times we need to have a pharmacoepidemiologist - no that is an epidemiologist; it’s a yah like a pharmacologist. You need a pharmacologist someone good with the terminology yes but someone looking at the finance, the health finance economic.

**3:10 The majority of the patients get their medicines from government so th…… (14791:15726) - D 3: KI_KM_MSH**

The majority of the patients get their medicines from government so the limited government budget to buy drugs has to go further and certain drugs even though the doctors are excited that this is the great drug but maybe they have something cheaper and also effective. So we always want to make sure that we review their request for addition in that light. So the few request that have, the very few requests that have been added to the medicine essential list have really been not really interrogated to that level, it’s more of oh this drug is familiar I have seen this ah that’s not too bad, so yah that will be the irrational side but it hasn’t been working well because we are not getting requests through the facility therapeutic committees because they don’t exist and also the few drugs that needs to be added, they don’t go through that rigour to check and confirm and capture. It’s just a case of oh well lets just include it.

**3:11 I think that is all that really exists in the country, we don’t have a…… (16064:17493) - D 3: KI_KM_MSH**

I think that is all that really exists in the country, we don’t have anything looking at the patients. Our focus has been more on the clinician and we are really seeing a situation where clinicians know what need to be done, but they probably don’t do it, but also most of our service provision is patient initiated if I were to put it that way. Where a patient will say ‘I have a headache, I need paracetamol or I need a green paracetamol or I need, I have a knee problem I need a small brown tablet’ so clinicians don’t actually because of well they claim that a lot of patients know to prescribe, they really don’t have time, when a patient says I want the blue tablet or the green tablet, they say oh ok if it works for you great right (laughs). So I think we have these forums that we have the processes and procedures for clinicians how to prepare the treatment, how to request for something to be removed, uhm we train nurses on the guideline, the pre-service, we develop job aids for nurses and providers. I think from a patients’ point of view or for the patient, we haven’t done much, so the patient for them they don’t really care about your guideline, this is what they want, what will work for them or it worked for some other auntie at church and when I had a headache at church she gave me this blue tablet, I also want the blue tablet even if they don’t know what the blue tablet is but they just want it (laughs).

**3:12 no we certainly not Okay we spend a lot of money on medicines and we d…… (17727:20965) - D 3: KI_KM_MSH**

no we certainly not Okay we spend a lot of money on medicines and we don’t see into the caption of the value of money. We use any drug at anytime, anywhere by anyone and partly because we are not as vigilant in what drugs do we use and how we use them. We are not as compliant with the treatment guideline. So no we are not okay we still need to do a lot, we still need to start having those pharmacy and therapeutics committees functioning at facility where the clinicians and service provider can sit together and talk about what are the issues and conditions that we are seeing at the facilities. Some of our hospitals, the majority of cases that they see are about primary health care cases you would find that in the A B C analysis, a hospital, a tertiary hospital has paracetamol in the top ten so already that tells you something that you know what’s going on. So we are doing something but we still need to do more, we still need to do more. We still need to do a lot of educating the prescribers, really showing them the value of complying with the treatment guideline. The value of making sure that we are using the essential medicine list and obviously not forgetting the patient, because really if we don’t focus on the patient every investment that we have put at addressing the clinical patterns that the prescriber practices - it will go to waste because the patient will be first and we have a, I think our citizens are very active in that if they go to a certain facility and they find that a certain product, they were not given a certain product - It can get to media and then it becomes a big story and gets political attention and you cannot say to the politician that the patient asked for antibiotics and the condition doesn’t need antibiotics. Politicians will say but it’s the patient’s right. It won’t fall, so we still need to bring in, I would have expanded to say the civil society into the rational medicine use that is not just professional, it’s also a civil action that the media and the journalist needs to know, so that you know, that there are products that you shouldn’t just use as when you want it. It is necessary to complete your course of antibiotics. It is necessary for you to use your painkillers as prescribed whatever so that as clinicians we are able to then to comply with our guideline but also we are able to make sure that the medicines that we bringing into the country are effective and the medicines that we are bringing into the country are cost effective, because the reason why we and obviously this is the main reason for developing the medical essential list to make sure that we have drugs that are effective, cost effective and also of high quality and with Swaziland being a small country, we struggle with the economy. So if we buy three different drugs for headache for example we would probably pay a lot of money because they are expensive in smaller volumes than if we were to buy one drug for headache for the whole country. So I think that’s what we need to look at when we talk of making sure that we comply with standard treatment guidelines. We make sure that everyone is ever close to them and they understand why we have them. So there is still a lot to be done.

**3:13 I think for me you know this is an interesting area of study and this…… (21157:21806) - D 3: KI_KM_MSH**

I think for me you know this is an interesting area of study and this is the first time really a study like this is being done in Swaziland. It will be more of looking forward to the report and recommendations because it will also help us structure our response both to the government, the partners like RFM external [NN2]and academia because we need to get academia actively involved in this but as much as they have experienced the guidelines we still need to make sure that they use them as the treatment guideline for the country. So I think for me we need to really appreciate that and we are looking forward to the outcome of the recommendation

**4:1 it is the using of ema-medicine in a cost effective manner ensuring, t…… (1033:1353) - D 4: KI_Lubombo region**

it is the using of ema-medicine in a cost effective manner ensuring, that there are no unnecessary expiries[ukuthi akubi nema-expires la-unnecessary] that are costly to the government in the country and also and also observing First-in-first-out when using medicines [ku observer labofirst in first out when using imitsi]

**4:2 yes and for the storerooms, the temperatures must be [kutsi abe] withi…… (1397:2217) - D 4: KI_Lubombo region**

yes and for the storerooms, the temperatures must be [kutsi abe] within the recommended temperatures for ema-medicines and for those small refrigerators [astoritshile lapho]. Following instructions and directions that they need on how medicines should be stored [ukuthi kubekeke kanjani], avoiding even overstocking and [nabo] understocking anytime, any [nabo-]polypharmacy for something we just have to order when prescribing for a patient you find that for instance okay you find a patient complaining with flu, you find that sometimes they just treat the symptoms instead of treating [lokutreater], okay giving the patient [lokutamsita] not to feel the whole pain bearing in mind medicines’ side effects and sometimes you find that you were just treating symptoms, like headache instead of using cold and flu, uyabona

**4:3 I think it’s ignorance, lack of knowledge, not following the standard…… (2539:2664) - D 4: KI_Lubombo region**

I think it’s ignorance, lack of knowledge, not following the standard treatment guideline or even trying to please the patient

**4:4 okay its user friendly, they are able to understand it too, it’s just…… (3238:3635) - D 4: KI_Lubombo region**

okay its user friendly, they are able to understand it too, it’s just that what makes them sometimes to commit mistakes is shortage of staff, and demotivation maybe. So many things are demotivating to them, to work in the same area/place everyday it’s so demotivating nje we just work for the sake of working but they are not doing it whole heartedly there is no dedication, they are just doing it.

**4:5 ok that one yes we do have, but unfortunately at the time it was being…… (3846:4027) - D 4: KI_Lubombo region**

ok that one yes we do have, but unfortunately at the time it was being introduced I was on leave I couldn’t make it there was another matron, that’s why I said you should talk to him

**4:6 facilities do need training number one and also if maybe there could b…… (4435:4612) - D 4: KI_Lubombo region**

facilities do need training number one and also if maybe there could be employment of a dispenser or pharmacist or pharmacy assistant or even the regional pharmacist or something

**4:7 maybe we need a regional pharmacist because most of the things that ar…… (5427:5908) - D 4: KI_Lubombo region**

maybe we need a regional pharmacist because most of the things that are pharmacy we don’t know them, we don’t know them - we are just nurses sometimes we look at the pharmacology for most of the things are much harder so that is why you find that even the patients are now complaining because they wait a long time and then sometimes you find that it’s the nurse who prescribes after seeing the patient then the patient has to wait for the very same nurse to go and get the medicine

**4:8 ) mmm okay the training, the employment of pharmacy personnel and (pau…… (6245:6477) - D 4: KI_Lubombo region**

) mmm okay the training, the employment of pharmacy personnel and (pause) and okay if they bring the order - to receive the things we require in time and also it has to be transparent as to what is available and what is not available

**4:9 yes I wanted to take the BCG to Mpolonjeni in the morning there was no…… (6699:6863) - D 4: KI_Lubombo region**

yes I wanted to take the BCG to Mpolonjeni in the morning there was no BCG. I was told at the facility stores supply that BCG is out of stock at the national level.

**4:10 it’s still a challenge, of knowing how do you go about if you have got…… (7831:7974) - D 4: KI_Lubombo region**

it’s still a challenge, of knowing how do you go about if you have got expired drugs in the facility, somebody can come and have different story

**4:11 the clinics, because I mean sometimes you find a person polishing his…… (8560:9158) - D 4: KI_Lubombo region**

the clinics, because I mean sometimes you find a person polishing his or her shoes gauze – just think how much that thing costs nge goldline – but if that person doesn’t know how much they cost you cannot blame her or him if they don’t know the cost of that thing, and also I think there should be security, I think it will be the best because even if we get the drugs, our storerooms are not lockable or sometimes they are locked but then you find that someone has taken the key and things just vanish from the shelves as to how we don’t know even at night there is no security at the facilities.

**5:1 I think using the medication appropriately to me means prescribing the…… (451:640) - D 5: shiselweni**

I think using the medication appropriately to me means prescribing the right medication for the right patient not giving like many antibiotics unnecessarily, I think that’s, what it is to me

**5:2 they have the standard treatment guideline? (1009:1052) - D 5: shiselweni**

they have the standard treatment guideline?

**5:3 We sent the order but now it hasn’t been delivered since July yet when…… (2525:3139) - D 5: shiselweni**

We sent the order but now it hasn’t been delivered since July yet when I try to call the matron who is responsible for the order, I get this “she is away”, after this I had to call the medical stores people to find out ukuthi whats their challenge and then they said that they know that they have got the order but then they haven’t been delivered, the person I talked to said they are still going to find out ukuthi where is the order now, so that’s how it happens to me it comes very late because they have to report to the sister and then the matron to the region if nothing is happening so it’s a long process

**5:4 there are challenges my dear, there are challenges like what I have go…… (3383:4020) - D 5: shiselweni**

there are challenges my dear, there are challenges like what I have got is that they don’t really communicate with each other these people in the clinic ukuthi “do you have such, such medicines and if you know have we are actually out of stock” then they do help each other if the other one has got a lot of big stock, even with the vaccines that’s what they do. If the other clinic runs out of vaccine they call each other to find out if they have it and then they help each other and share the prescription. We have challenges, challenges we do have, and we do have. I would be lying if I say we don’t have challenges with the stock out

**5:5 I think they can benefit from it because… yah I think they can benefit…… (5091:5358) - D 5: shiselweni**

I think they can benefit from it because… yah I think they can benefit a lot because we don’t have the pharmacist, this was what the ministry had promised that they will, they are planning to send the regional pharmacist who will be doing the supervision in the region

**5:6 exactly we do need a pharmacist or a pharmacist assistant, something l…… (6167:6687) - D 5: shiselweni**

exactly we do need a pharmacist or a pharmacist assistant, something like that, someone who would be looking at the pharmacy because it is really, really too much for the nurses because all the clinics, their maximum there are three nurses and if there is a sister then the sister is the forth one but nje most of the clinics there are three nurses can you imagine the work really a lot of work now there is this thing that you enter, they type, do check history prescribing and going to dispense and sometimes yah it is…

**5:7 I wasn’t understanding okay I knew ukuthi e-clinic they have shortages…… (10206:10673) - D 5: shiselweni**

I wasn’t understanding okay I knew ukuthi e-clinic they have shortages, shortage of stuff but until I came in here, I was once invited to one of the clinics but when I got there, I asked myself how are they operating when they are three in the clinic and when there is so much work, you know us TB clinic, ANC, drug use, family planning, they are just three people but now I am here in it and maybe these workshop which they keep calling them on left right and centre
