# Supplementary material for: Health system actors’ perspectives of prescribing practices in public health facilities in Eswatini: A Qualitative Study
Source: PLoS One. 2020 Jul 9;15(7):e0235513. doi: 10.1371/journal.pone.0235513 (PMC7347100; doi:10.1371/journal.pone.0235513)
Supplement: S5 File — (DOC) [file pone.0235513.s008.doc]

# Project: Baseline RMU Facilities Analyses

Report created by NONDUMISO on 2/2/2018

**Quotation Report**

All (327) quotations

**8:1 basically we had a challenge at the facility and at the country at lar…… (431:753) - D 8: HH_dvk**

basically we had a challenge at the facility and at the country at large on the rational use of medicine, it’s not a very big strong hold and there are no strengthening guideline protocols that are in place unfortunately to make sure that there is continuous monitoring of rational use of medicines in Swaziland as a whole.

**8:2 So generally at the facility we do have a therapeutic committee that i…… (755:833) - D 8: HH_dvk**

So generally at the facility we do have a therapeutic committee that is working

**8:3 We do deliberate a lot in terms of the usage of medicines for instance…… (836:984) - D 8: HH_dvk**

We do deliberate a lot in terms of the usage of medicines for instance but on the ground it’s not very much practiced, that is the challenge we have.

**8:4 from the perspective of different prescribers because we have got ehm…… (1148:1401) - D 8: HH_dvk**

from the perspective of different prescribers because we have got ehm varied prescribers we have got nurses who are also prescribing medicines, generally within the nursing profession, the practicing is poor should I put it that way, it is totally poor.

**8:5 there is a general consecious feeling, whether it’s a storage feeling…… (1437:1565) - D 8: HH_dvk**

there is a general consecious feeling, whether it’s a storage feeling that the patient should go with quite a number of medicines

**8:6 there also is also pressure from the patient that require a lot of var…… (1571:1952) - D 8: HH_dvk**

there also is also pressure from the patient that require a lot of variety of medicine. Patients are not satisfied if they are going away with maybe three types of medicines when they leave the facility, ah maybe they have a flu or headache or maybe a simple injury, you discover that they are not satisfied once they give, if you just give them one or two medicines, they want many

**8:7 So at the end of the day they give pressure to the prescribers, that “…… (1955:2143) - D 8: HH_dvk**

So at the end of the day they give pressure to the prescribers, that “can I have more” at the end of the day they are prescribed medicines that are not in the cabinet for their own problems

**8:8 there is no resourcement within the nursing prescribing tendencies lik…… (2201:2303) - D 8: HH_dvk**

there is no resourcement within the nursing prescribing tendencies like who should prescribe as a nurse

**8:9 no training in the way they prescribe and write their prescription. (2373:2440) - D 8: HH_dvk**

no training in the way they prescribe and write their prescription.

**8:10 The treatment is according to symptoms, so if you are sneezing you get…… (2557:3140) - D 8: HH_dvk**

The treatment is according to symptoms, so if you are sneezing you get items for sneezing, if you are coughing you get something for coughing and if you have an itchy eye you get something for the itchy eye. Itch nose you get something for the itchy nose, if you have irritating throat they give you something for irritating throat, host continuous like that, instead of making a proper diagnosis what is the proper diagnosis, maybe the person has got one diagnosis which is flu or which is tract infection. Just treat the disease, what do they do, each symptom with each medication.

**8:11 That is the challenge we have. So the weakness there is training and n…… (3141:3463) - D 8: HH_dvk**

That is the challenge we have. So the weakness there is training and not only training its again changing the culture and the practice and this practice and culture I think it emanated long time back. It has been there, training practices as well that you just need to prescribe as many medicines as possible to the client.

**8:12 They don’t look at the detrimental effects of those medicines and thei…… (3465:3798) - D 8: HH_dvk**

They don’t look at the detrimental effects of those medicines and their interaction with the and the kind of medicine as well, whether the patients who are going to take the medicine and whether the person is taking the medicine from another facility because of what we see definitely our clients they visit regularly at our facility,

**8:13 they also have visit also visit nearby facilities, the government fac…… (3834:4228) - D 8: HH_dvk**

they also have visit also visit nearby facilities, the government facility the nearby clinics it’s not only the nearby clinics they also have to visit the nearby health centres. They interchange move from one corner to corner, not only that they also go to the private sectors and it’s not only that they go to private sectors only, and they also go to traditional healers within the community

**8:14 So you see our practices, as a facility, as the prescribers we don’t r…… (4231:4769) - D 8: HH_dvk**

So you see our practices, as a facility, as the prescribers we don’t really take that into consideration. The side effects of those medicines the interaction of those medicines be it the other medicines that they are collecting, there is no collective purpose of trying to investigate where these clients visit as well before you prescribe the medicines So there is just a generally tendency to prescribe as many medicines as possible it’s a culture is that is doing this then they are the prescribers, the nursing profession in particular

**8:15 Then you go to the doctors as we (pause) some doctors okay for instanc…… (4772:5062) - D 8: HH_dvk**

Then you go to the doctors as we (pause) some doctors okay for instance, for example here at Dvk, for this year we have, doctors that come from different parts of Africa, or the world or they would be trained in various parts of the world in China, they have been trained at the DRC.

**8:16 Their competence is not tested, in terms of prescribing or their knowl…… (5064:5557) - D 8: HH_dvk**

Their competence is not tested, in terms of prescribing or their knowledge in the disease pattern that are affecting the Southern region as well as the patterns again that affects the local region. They struggle in the early days to uh to understand the disease pattern that we do have so at the end of the day they just prescribe anything maybe according to whatever they have been trained, so there is also a general problem of not following the treatment guideline of which they are there.

**8:17 But the guidelines are not followed. (5558:5593) - D 8: HH_dvk**

But the guidelines are not followed.

**8:18 the guidelines have been there yah; they have been there for years. I…… (6647:7117) - D 8: HH_dvk**

the guidelines have been there yah; they have been there for years. I am sure the last update was 20 if I am not mistaken was from last 2012 to 2013 and then no guidelines are there. but people just, preferable even if you visit any prescribing table or the prescribing room you won’t see any guideline there even the essential medicine list is still there but you won’t even see, those document, people just put them in their drawers or in their rooms under the pillows

**8:19 do you see any improvements in terms of the prescribing patterns? Info…… (7289:7438) - D 8: HH_dvk**

do you see any improvements in terms of the prescribing patterns?

Informant: I would want to say not at all, it look like the graph is going down yah.

**8:20 the tendency is you see we have a problem that we talk maybe in a ther…… (7469:7915) - D 8: HH_dvk**

the tendency is you see we have a problem that we talk maybe in a therapeutic committee today hope that people will bring sort of change then after that the graph just go down. People change into their old practices that’s why I said it’s a culture, a culture is difficult to change for someone who is maybe they have been taught at school that this is what they are supposed to do and so you know trying to change that culture is a long process.

**8:21 I would want maybe the government in particular the cabinet of pharmac…… (8185:8907) - D 8: HH_dvk**

I would want maybe the government in particular the cabinet of pharmacy to enforce and strengthen this area of rational drug use. There is one other area that is draining a lot of resources as well from the country and it is very detrimental to the population the patient themselves knowingly and unknowingly people are suffering from, some of the medicine that they are getting, they are the medicines that are having problems in their bodies as well because of these huge numbers of items that they collect from the facility so if the government doesn’t look to it and drag their feet I think we do have a challenge I feel the department of pharmacy should really try to import and continuously monitor the improvement.

**8:22 The challenge that we have is talk of something today, there is no con…… (8908:9067) - D 8: HH_dvk**

The challenge that we have is talk of something today, there is no continuous monitoring to see whether those things are happening on the ground or they are not

**8:23 Then training is very important and continuous training of prescribers…… (9187:9461) - D 8: HH_dvk**

Then training is very important and continuous training of prescribers as a whole at the facility we feel that we need support from the top managers okay the top managers are not there to sit in the office they are supposed to be hands on, to come to the ground be with us

**8:24 because once you, even if you try and enforce some of these policies…… (9463:10372) - D 8: HH_dvk**

because once you, even if you try and enforce some of these policies alone they will not listen people don’t listen particularly when you are a foreigner people just up to you because we know in Swazi we know that we just treat people like this but that is not it we are trying to help people. We are not saying don’t give medicines as a whole but we are saying don’t give a lot of unnecessary medicines to people because those medicines cause problems in the body and you as nurses you don’t know exactly what they cause on the body. The advice if they are just referred who knows maybe they are really caused by the medicines that they are taking yah so I feel that there is need for the top managers to come down to the facility not to stay in the office and operate from there but they should be on the ground helping us and enforce. People who are on the ground they should feel that we have a backup yah

**8:25 not really because we haven’t had quite a number of maybe long term st…… (10549:11019) - D 8: HH_dvk**

not really because we haven’t had quite a number of maybe long term stock out and even if we do have some stock out here and there. It’s not a reason to maybe give ten medicines for one treatment for a person it’s not a reason for fifty medicines in one time, you can make your own substitute that goes along the lines depending on the guideline and okay that let me take this one and this one. But its not a reason to say let me give many medicines per person irrational

**8:26 yah the culture needs to change and the people should have the spirit…… (11277:11583) - D 8: HH_dvk**

yah the culture needs to change and the people should have the spirit to change the managers top managers should also be available hands on as well, the department should have policies and enforce those policies so that at least people follow the guideline basically those are the things we need to work on.

**9:1 i-patient inembizo uyiphendule kahle so nokho kuyasita le-patient abew…… (634:1213) - D 9: HH_ekuf**

i-patient inembizo uyiphendule kahle so nokho kuyasita le-patient abewati umhlobo wamaphilisi, okuthi athathwa kanjani nokubekwa kwawo ukuthi alapheke kahle, kuphinde kufike nalabo abefasilithi ukuthi i-stock sethu sihlale sikahle ngoba siyathola ukuthi kungaba labantfu abangaki sibanika according to ukugula kwalabantfu, sibanika amaphilisi amangaki until we are finished so kuyafika kahle kumbe sokushesha kuphela kumbe kunengi kakhulu ku-stock bese u hulumende ngabe lithi yi-central medical stores ukuthi nayo iyakhona ukuyenta i-management ekahle kuma-drugs with no westage

**9:2 cha, ngombono wami hayi angikatsho cha, yebo ngokubuka kwami si-prescr…… (1327:1742) - D 9: HH_ekuf**

cha, ngombono wami hayi angikatsho cha, yebo ngokubuka kwami si-prescriber ngendlela ekahle esafunda ngayo langendlela esithi mhlawumbe masiye ema-workshops sifundiseke ngayo even though lengingakutsho ukuthi masifika, it was a new facility and they were only two new nurses so besisite i-experience enganani of managing stock and drugs but nje besiyenta ngalendlela esafunda ngayo si-prescriber using ama-guidelines

**9:3 okay first time I got to know about the guideline was when it was intr…… (2456:2564) - D 9: HH_ekuf**

okay first time I got to know about the guideline was when it was introduced at university, we found it there

**9:4 talking about update, it needs it because there are somethings that ar…… (3163:3330) - D 9: HH_ekuf**

talking about update, it needs it because there are somethings that are not there yet there are patient who come here for treatment of conditions which are not included

**9:5 yes because we do have some medicines out there we know what they are…… (3669:4033) - D 9: HH_ekuf**

yes because we do have some medicines out there we know what they are good for, yes and let’s say that that illness requires ukuthi ngikunike ama-antibiotics, utabe use with understanding that you have ngama-antibiotics ukuhlukana kwawo yiwaphi a-bad for this lokuthi ayelapha kanjani ma ulobuhlungu ukuthi yona nje but vele kude okubhalwe phansi ukuthi umnika aphi

**9:6 we just talk to our matron; she is the one who usually… (5645:5699) - D 9: HH_ekuf**

we just talk to our matron; she is the one who usually…

**9:7 not exactly? So would you say it’s something that you need at the faci…… (5946:6020) - D 9: HH_ekuf**

not exactly? So would you say it’s something that you need at the facility?

**9:8 because we have like I graduated in 2016 in the university, so at univ…… (6093:6452) - D 9: HH_ekuf**

because we have like I graduated in 2016 in the university, so at university you don’t get to learn about those things we just catch up here at work, so sometimes there may be somethings that you shouldn’t even do but you don’t know those things so you just learn from your older colleagues but unfortunately here we don’t have older colleagues at the facility

**9:9 just that sometimes we do run out of stock and we are never told, we d…… (7078:8077) - D 9: HH_ekuf**

just that sometimes we do run out of stock and we are never told, we do order but we never get our orders and never told if the order is not there or it is coming it’s on its way. Last month we are in October now and we didn’t get our September and August order, it was supposed to come. So sometimes we feel safe to order stock for three months and if we run out of some medicines we ask from our neighbouring facilities, and sometimes you find that they don’t have what you also don’t have. We have to just write a prescription and then ask our clients to go to pharmacies for or some other hospital and if they can afford they can just pick up their prescription and go to the hospital. We ask them so nathi i-strain to ask the client; we know that some of them don’t have the money to go to the hospital in town or pharmacies, yes so that kind of challenge is yet to come across right now for instance we don’t have hypertension drugs, drugs that came right now, we don’t have hyper tension drugs

**9:10 maybe it could be nothing maybe there could be some inservice training…… (8802:9039) - D 9: HH_ekuf**

maybe it could be nothing maybe there could be some inservice training on the uses of medicine, the duration of the medicines, I don’t know maybe from central medical stores, so that we can be on the same page about the drugs in hospitals

**10:1 RMU is avoidance of polypharmacy, prescribing of drug of choice for ea…… (416:575) - D 10: HH_Mkz**

RMU is avoidance of polypharmacy, prescribing of drug of choice for each and every patient or condition, and avoidance of prescribing a cocktail of antibiotics.

**10:2 Not all prescribers are, but we talk to those that are not. The pharma…… (658:1071) - D 10: HH_Mkz**

Not all prescribers are, but we talk to those that are not. The pharmacy personnel call that prescriber when they receive a prescription and feel there is some irrational use of medicines to discuss the issues. However, a lot of times the pharmacy staff come across challenges when they do this as some of the prescribers then feel undermined, offended, and most of the time they refuse to change the prescription.

**10:3 times the pharmacy staff come across challenges when they do this as s…… (887:1071) - D 10: HH_Mkz**

times the pharmacy staff come across challenges when they do this as some of the prescribers then feel undermined, offended, and most of the time they refuse to change the prescription.

**10:4 Yes, pharmacy is doing our best to properly label and counsel our pati…… (1241:2018) - D 10: HH_Mkz**

Yes, pharmacy is doing our best to properly label and counsel our patients on the medicines that they are taking. This also depends on the age and literacy of the patient. We do have a challenge with language though as we both are not fluent in SiSwati, but having been in practice for a long time we are able to explain and ensure that the patient understands. We also have grannies who may not understand the labels written in English, in such instances we really make it a point to explain so that they understand their medicines, why they are taking them and how they are to take them. It’s just that you have come on a Tuesday and we are not very busy, if the patients were here you could ask them. They would attest to the fact that we really explain the medicines to them

**10:5 However, I have already said that some prescribers are not prescribing…… (2023:2225) - D 10: HH_Mkz**

However, I have already said that some prescribers are not prescribing rationally. If irrational use is done from the facility there is no way that a patient will go home and correct that irrational use.

**10:6 We have the national STG/EML but I must say that prescribers are not u…… (2345:3262) - D 10: HH_Mkz**

We have the national STG/EML but I must say that prescribers are not using it at all. We have had some meetings in the past where I would ask if prescribers, particularly nurses, are using the guideline or if they have at least read through it, and these prescribers would say no.

We also have a PTC that is partly functional. My expectations for this committee would be to discuss RMU, challenges, and prescriptions. I do not get to attend these meetings, my colleague does and I have said to him I also want to attend in future. When I go through the PTC minutes, the committee is not discussing any of the issues that I feel should be tackled by the PTC. Also, I would expect that as a facility we have our own formulary derived from the National STG/EML, however this is not the case though the national guideline is available. The Senior Medical Officer (SMO) has promised to address these issues in future meetin

**10:7 None have been happening. The SMO has however promised that clinical m…… (3416:4064) - D 10: HH_Mkz**

None have been happening. The SMO has however promised that clinical meetings will soon be starting. I think as a team we are capable of training amongst ourselves but we would also need support from an outsider. In this facility both of us are Pharmacy technicians, it would help to also have a Pharmacist come to train us. Also, maybe the staff would better listen to an outsider than they would listen to us. In my opinion, people are shy to open the guidelines in front of the patient. They want to pretend as if they know and that is when they get lost. If we were using the guidelines properly, we would not be talking of irrational drug use.

**10:8 In my opinion, people are shy to open the guidelines in front of the p…… (3828:4063) - D 10: HH_Mkz**

In my opinion, people are shy to open the guidelines in front of the patient. They want to pretend as if they know and that is when they get lost. If we were using the guidelines properly, we would not be talking of irrational drug use.

**10:9 I think there needs to be monthly in-service trainings, promoton of u…… (4184:4345) - D 10: HH_Mkz**

I think there needs to be monthly in-service trainings, promoton of use of guidelines, continued discussions between pharmacy and prescribers whenever necessary.

**11:1 I think it’s giving the correct medication for a specific diagnostic t…… (299:507) - D 11: HH_Mshin**

I think it’s giving the correct medication for a specific diagnostic that has been identified in the patient. Medication that will quickly take care of any kind of diagnosis, come up with the, it should be it.

**11:2 we are talking about picking the correct amount of medication at the c…… (662:788) - D 11: HH_Mshin**

we are talking about picking the correct amount of medication at the correct time interval and initiating the proper treatment.

**11:3 I think we are trying our best. I can’t say maybe we are 100% because…… (1034:1301) - D 11: HH_Mshin**

I think we are trying our best. I can’t say maybe we are 100% because in some instances you can diagnose someone differently than the way someone else would but we are really trying our best and the time for giving the right medication to the right patient is limited.

**11:4 you quickly and dispense knowing it can heat up. (1332:1379) - D 11: HH_Mshin**

you quickly and dispense knowing it can heat up.

**11:5 we have the standard treatment guideline (1796:1835) - D 11: HH_Mshin**

we have the standard treatment guideline

**11:6 yes we have one, I uhm where is it, yah ingale I think so (2014:2070) - D 11: HH_Mshin**

yes we have one, I uhm where is it, yah ingale I think so

**11:7 we try our best to adhere to the guideline, sometimes I am not sure be…… (2251:2503) - D 11: HH_Mshin**

we try our best to adhere to the guideline, sometimes I am not sure because for me, I am a new nurse so most of the time I carry the guideline everywhere, and I find myself adhering to the guideline if I am not make sure of what to prescribe the patient

**11:8 by the people from the pharm care and health ministry (2853:2905) - D 11: HH_Mshin**

by the people from the pharm care and health ministry

**11:9 yes if only maybe there could be somewhere maybe written in charts so…… (3124:3366) - D 11: HH_Mshin**

yes if only maybe there could be somewhere maybe written in charts so that you can just easily flow. Because they are a book you have to read, so maybe if they were using at least some of charts you just go to the page quickly and you are done

**11:10 besides the guideline sometimes we do have our matron coming here to r…… (3755:3859) - D 11: HH_Mshin**

besides the guideline sometimes we do have our matron coming here to refresh us giving us a fresh concept

**11:11 sometimes we even have meetings amongst ourselves so to assist each ot…… (3914:4159) - D 11: HH_Mshin**

sometimes we even have meetings amongst ourselves so to assist each other especially with the conditions we see on a daily basis like the common care conditions in our area or what would you do for a child like that and then we assist each other.

**11:12 because we believe there they have more stock than us because we are j…… (4567:4690) - D 11: HH_Mshin**

because we believe there they have more stock than us because we are just a small clinic there are drugs that we don’t have.

**11:13 I would say we would love for our head pharmacy to give us most specif…… (5043:5394) - D 11: HH_Mshin**

I would say we would love for our head pharmacy to give us most specific antibiotic like for example for skin conditions, like here we see children with very different skin conditions, but we don’t have those antibiotics like we don’t have so we just have to give completely different medicine, so we would love to have those more specific antibiotics.

**11:14 it would be a very good initiative especially in training vele most of…… (5822:5980) - D 11: HH_Mshin**

it would be a very good initiative especially in training vele most of us we are new nurses here in the facilities yah I think so because we are young ones now

**11:15 yes through our committee matron, we don’t meet with them vele but the…… (6294:6400) - D 11: HH_Mshin**

yes through our committee matron, we don’t meet with them vele but the matron would liaise with them for us

**11:16 this is the current situation that we in nje right now we are out of m…… (6709:6787) - D 11: HH_Mshin**

this is the current situation that we in nje right now we are out of medication

**12:1 do have the new conditions that are coming up, they are definitely are…… (1728:1894) - D 12: HH_NatB**

do have the new conditions that are coming up, they are definitely are…, there are conditions that needs to be included in the guideline or maybe reviewed and so forth

**12:2 we don’t have this in the clinic but if we have any queries, if we hav…… (2551:2747) - D 12: HH_NatB**

we don’t have this in the clinic but if we have any queries, if we have go maybe to the central medical stores for ordering we are open to ask them anything that we don’t …, we are not sure of, yes

**12:3 yes sometimes you find that they are very busy and so forth but they d…… (2877:3035) - D 12: HH_NatB**

yes sometimes you find that they are very busy and so forth but they do help us whenever they have time to sit down and discuss with us if we have any queries

**12:4 yah sometime, two weeks back we had somebody who came here, she wanted…… (3192:3404) - D 12: HH_NatB**

yah sometime, two weeks back we had somebody who came here, she wanted to see how we were going about, like maybe about our storage, place any question we were allowed to ask whatever we did not know or understand

**12:5 well they don’t come on a regular basis, especially here; maybe it’s a…… (3434:3566) - D 12: HH_NatB**

well they don’t come on a regular basis, especially here; maybe it’s a bit outdoor maybe they are short stuffed and busy and so forth

**12:6 mentorship definitely, we definitely need them, here like I said we ar…… (4130:4294) - D 12: HH_NatB**

mentorship definitely, we definitely need them, here like I said we are so far, and we are only two or three nurses or so but this year there is no one who came here

**12:7 with stock out we try not to because we go to these private pharmaceut…… (4599:4749) - D 12: HH_NatB**

with stock out we try not to because we go to these private pharmaceuticals pharmacies whenever we need medicines that we can’t get from the government

**12:9 to medicine not really, no but our main concern is the area that we ar…… (5445:5712) - D 12: HH_NatB**

to medicine not really, no but our main concern is the area that we are working in, our patients are very poor they can’t afford and buy medicines. If we prescribe something for them they tell you make we can’t afford, they are relying on government too and that is a…

**12:10 yah the problem is that we don’t have a pharmacy assistant of any sort…… (6045:6325) - D 12: HH_NatB**

yah the problem is that we don’t have a pharmacy assistant of any sort it’s a bit of a problem because whatever maybe queries we have, we have to go to central medical store and sometimes they are too busy, they don’t have time to discuss anything with us. It is a bit of a problem

**13:1 I think it’s the correct usage of medicines, like using the correct do…… (388:506) - D 13: HH_ngow**

I think it’s the correct usage of medicines, like using the correct dose and according to… prescribing according to age

**13:2 we do have guidelines, in case we find out that we are stuck and then…… (831:1032) - D 13: HH_ngow**

we do have guidelines, in case we find out that we are stuck and then when we are not sure of what to prescribe. We have guidelines for generally for all the other diseases that we meet at the facility

**13:3 mmm on user friendly yes it is but most of the conditions you find tha…… (1338:1569) - D 13: HH_ngow**

mmm on user friendly yes it is but most of the conditions you find that the prescription that they recommended treatment for the conditions most of the most of those medication you find that we don’t have them, they are not supplied

**13:4 the reason is that we are ordering but even if we order them be we don…… (1757:1976) - D 13: HH_ngow**

the reason is that we are ordering but even if we order them be we don’t get them back. There are certain medicines or pills that like I have said we need them for the community but are not recommended for clinical level

**13:5 but the problem with central medical stores is that they don’t write t…… (2968:3304) - D 13: HH_ngow**

but the problem with central medical stores is that they don’t write the feedback to tell us if there is sock out of any particular medicines. They don’t write on the order form like they do with the non-essential medicines, we order separately on the supply stores, so if they have something out of stock they write this is out of stock

**13:6 internally we don’t have but we address those issues with people who c…… (3771:4066) - D 13: HH_ngow**

internally we don’t have but we address those issues with people who come for like visits like they usually come for stock taking in this day and on the other days they will be checking the drug form, checking the conditions in the drug form, that’s the system that we have to address such issues

**13:7 we did receive it from the central medical stores, I think sometime la…… (4255:4331) - D 13: HH_ngow**

we did receive it from the central medical stores, I think sometime last year

**13:8 like I mentioned before some of the stock, some of the drugs that we n…… (4833:5310) - D 13: HH_ngow**

like I mentioned before some of the stock, some of the drugs that we need are not supplied, so sometimes you find yourself giving a person like I can make an example, you find yourself giving a child something that you are not supposed to give just because maybe the child is vomiting and there is nothing to stop the vomiting and then we decide to give the child who is less than 3years adult medicine, so in cases like that we find ourselves prescribing outside the guideline

**13:9 on the challenges that we do have when it comes to appropriate use of…… (5883:6466) - D 13: HH_ngow**

on the challenges that we do have when it comes to appropriate use of drugs. I think that it can be a suggestion, just to appeal with the central medical stores that at least they can ask us the conditions that we usually have like the diagnosis and just to feel that facility X usually they have these conditions so they do need this because you find that we have a lot of cases but when there is nothing like cough syrup or cough mixture for children and you can imagine the trouble. You end up giving the child something that is not supposed to be given to a child so help me there

**14:1 correct use of medicines which means the right medicines to the right…… (436:678) - D 14: HH_ppk**

correct use of medicines which means the right medicines to the right patient at the right dose considering weight in most instances and the complete course as well as the patient as well taking the medicines for the recommended period of time

**14:2 the challenges of say stock out when we do not have what is appropriat…… (823:1091) - D 14: HH_ppk**

the challenges of say stock out when we do not have what is appropriate you go for the next best thing and in that instance it’s not the correct medicine for that particular condition, yes maybe if we had everything available, I would say yes we do prescribe rationally

**14:3 we have umm, some will call a drug worksheet which is a list of drugs…… (1258:1691) - D 14: HH_ppk**

we have umm, some will call a drug worksheet which is a list of drugs that are available in the facility which people can prescribe according to and we continuously tell them what we have available and what we have not available. In any case if there is a condition that requires something outside our hospital for example then that way we will try by all means to get it and prescribe appropriately if there is nothing then… (Laughs)

**14:4 mmm I would say yes and no. the treatment guideline is not comprehensi…… (1919:2194) - D 14: HH_ppk**

mmm I would say yes and no. the treatment guideline is not comprehensive, for instance I would say they are not meant for hospitals as well so in some instances we follow them but we are at a hospital level and we require more than what is in the standard treatment guideline.

**14:5 mmm maybe just to track our own history when they started forming or m…… (2443:2846) - D 14: HH_ppk**

mmm maybe just to track our own history when they started forming or making the guideline, various stakeholders were involved, I am assuming the pharmacist who was here by then who was actively involved in that by that time, some of the doctors who may or may not be available around, they were involved in that, so it was a disciplinary committee or forum that sat down and came up with that guideline

**14:6 I closely stick to the SAMF, the South African Medicine Formulary, the…… (3966:4132) - D 14: HH_ppk**

I closely stick to the SAMF, the South African Medicine Formulary, there is a lot that you learn from the formulary; I was educated in South Africa so that’s the thing

**14:7 I would say it’s functional but it’s not as effective or as functional…… (4393:4671) - D 14: HH_ppk**

I would say it’s functional but it’s not as effective or as functional as it once was or we would want it to be… for instance we have had a PTC meeting for a while, ideally we would be having one every three or four months as a facility, once every three months in any case, yes

**14:8 we need to be all participational in the PTC meetings for instance I a…… (4884:5685) - D 14: HH_ppk**

we need to be all participational in the PTC meetings for instance I am not sure if I am just trying to come up with a reason myself but it’s coming up as a pharmacy thing, which is to be driven by pharmacies, with the cutting medicine out of stock and which for my case, I think it’s not, it shouldn’t be entirely on that. The PTC is functional on team work, it shouldn’t be like informing them, and such and such is out of stock and what is available on stock. It should be looking at say for instance we are having a challenge in treating hypertension, you know, what is it that we are doing wrong? This is what we can improve on. The drug thing will come up, are we having enough, do we have the correct medicines or maybe do we need to stock another anti-hypertension drug that is not available in

**14:9 we have clinical meetings every week, different departments will come…… (5817:6156) - D 14: HH_ppk**

we have clinical meetings every week, different departments will come up and discuss different cases, then from those clinical meetings, that’s when I see if the concern or the challenges brought up, then it is taken to the PTC which will then sort of come up with the last word which is then dropped on how we can handle that as a hospital

**14:10 I would say no because patients for instance they come to the hospital…… (6294:6470) - D 14: HH_ppk**

I would say no because patients for instance they come to the hospital, if they come to the hospital they expect to get something even if they do not need the drugs or medicines

**14:11 will feel like no I haven’t been treated and they will then go away di…… (6478:6834) - D 14: HH_ppk**

will feel like no I haven’t been treated and they will then go away dissatisfied and ourselves as prescribers we want to keep our patients satisfied, we don’t want our patients going around complaining that we do not give them medicines “yoh udokotela didn’t give me any antibiotics, I was looking for an antibiotic” so basically I would say no they don’t.

**14:12 I would say no because patients for instance they come to the hospital…… (6294:6834) - D 14: HH_ppk**

I would say no because patients for instance they come to the hospital, if they come to the hospital they expect to get something even if they do not need the drugs or medicines, they will feel like no I haven’t been treated and they will then go away dissatisfied and ourselves as prescribers we want to keep our patients satisfied, we don’t want our patients going around complaining that we do not give them medicines “yoh udokotela didn’t give me any antibiotics, I was looking for an antibiotic” so basically I would say no they don’t.

**14:13 training in the hospital, yes but we hardly move up, we don’t say to o…… (6946:7267) - D 14: HH_ppk**

training in the hospital, yes but we hardly move up, we don’t say to our own patients for instance, it’s something that we have been actively involved in besides telling them his is how you use the medicines and the antibiotics you complete the course, you take it once three times a day till you complete, beyond that no.

**14:14 first of all I think we need to have like a consistent supply of drugs…… (7400:8513) - D 14: HH_ppk**

first of all I think we need to have like a consistent supply of drugs, they need to make the guidelines that are conducive for our hospital for instance, come up with maybe treatment guidelines for us as hospitals maybe not necessarily like Mbabane clinic or other facilities but one that we would design ourselves and will be able to follow and speak to because the current one maybe we have reasons to say that it was designed by people who were sitting at Orion, and who don’t know what is happening at the facilities so I think if we could have one that is localised to the hospital and also we need to educate or maybe train people and constantly upgrade ourselves on be it conditions and also on medicines because we have new people coming soon, maybe there is a new nurse coming here from school, he is not aware of what is happening on the ground, we have new people joining us, we have new doctors from different areas or even different countries coming in who are not aware with the system in the country so maybe education would really help and education for the patients as well on drugs and medicines

**14:15 maybe just another thing to add as well, the… within the country or ma…… (8671:9023) - D 14: HH_ppk**

maybe just another thing to add as well, the… within the country or maybe for the pharmacy sector there a continuous courses, that they would continuously help us upgrade and be aware of things and remind us as well of some of the drugs that we are using how to manage them, how to treat them. So that’s the thing that is also a big gap for the country.

**15:1 I don’t know whether (laughs) I will give the correct answer but I thi…… (568:712) - D 15: HH_sig**

I don’t know whether (laughs) I will give the correct answer but I think it’s to prescribe the right medicine for the right illness of the client

**15:2 we are, we are, like nje there are few problems because they were pres…… (816:1071) - D 15: HH_sig**

we are, we are, like nje there are few problems because they were prescribing a lot of medicines for the patients maybe five or six drugs and we said no guys, this is too much, I mean three drugs will be enough for one client not eight, ten drugs you know.

**15:3 I think they are prescribing a lot of drugs to impress some patients,…… (1073:1311) - D 15: HH_sig**

I think they are prescribing a lot of drugs to impress some patients, when I ask them, they say “no sister, you know these clients were really given two, and they said no I want this for rubbing? The patients will tell them what they want.

**15:4 oh okay you were talking about that one, yah we do have that one (1797:1860) - D 15: HH_sig**

oh okay you were talking about that one, yah we do have that one

**15:6 I think so of course we need it; definitely I think it’s vital especia…… (3599:3690) - D 15: HH_sig**

I think so of course we need it; definitely I think it’s vital especially to prescribe drugs

**15:7 yah my main, main problem is that we don’t have a pharmacy assistant s…… (3876:4302) - D 15: HH_sig**

yah my main, main problem is that we don’t have a pharmacy assistant so we use the orderlies but the nurses just try to train them you know as the name of the drugs, how to give those drugs, but we have a challenge because we don’t have stuff, we have got only two nurses, so it’s impossible for them to prescribe for the client and go to the pharmacy to give the drugs, so we are using the orderlies to dispense the medication

**15:8 it’s the nurses who give the information before the patients go and co…… (4542:4626) - D 15: HH_sig**

it’s the nurses who give the information before the patients go and collect the drugs

**15:9 yah the orderlies are just to give the drugs and tell that you take it…… (4664:4836) - D 15: HH_sig**

yah the orderlies are just to give the drugs and tell that you take it two times a day for three days, but the information is which is given from the nurses’ side is enough.

**15:10 mmm if we have only a pharmacy assistant. We need one because okay thi…… (4914:5120) - D 15: HH_sig**

mmm if we have only a pharmacy assistant. We need one because okay thina we were only trained when we were at school about how much you give, it’s not something where you just mix to produce the exact copies

**15:11 the other challenge we have is like now we don’t have most of the drug…… (5333:5486) - D 15: HH_sig**

the other challenge we have is like now we don’t have most of the drugs we have to try and call other clinics to give us these drugs if they have any also

**15:12 sometimes what we order, we are not getting it sometimes they are givi…… (6028:6134) - D 15: HH_sig**

sometimes what we order, we are not getting it sometimes they are giving us less stock from what we ordered

**15:13 yah they cut the number, yah they cut the number like maybe at times y…… (6351:6618) - D 15: HH_sig**

yah they cut the number, yah they cut the number like maybe at times you order ten bottles of a certain medicines and the give you five, sometimes they write OS and you won’t be having enough stock coming in compared to the number of patients that need that medication

**15:14 the other thing maybe it’s the stuff, sometimes maybe we don’t order e…… (6650:6792) - D 15: HH_sig**

the other thing maybe it’s the stuff, sometimes maybe we don’t order enough, sometimes you find that they are not having it for the whole month

**15:15 yes and we have not been trained you know, it’s an on job thing where…… (6997:7092) - D 15: HH_sig**

yes and we have not been trained you know, it’s an on job thing where we figure it out ourselves

**15:16 okay another thing is that maybe the pharmacy department should be goi…… (8259:8410) - D 15: HH_sig**

okay another thing is that maybe the pharmacy department should be going around maybe once in a month to see whats really going on, on the pharmacy side

**15:17 I think that maybe can help, so that we talk to them about the stock c…… (8444:8550) - D 15: HH_sig**

I think that maybe can help, so that we talk to them about the stock challenges and safety problems we have

**15:18 so that we know, we come didn’t come here for pharmacy stuff, we don’t…… (8581:8804) - D 15: HH_sig**

so that we know, we come didn’t come here for pharmacy stuff, we don’t know anything, who do we ask when we have problems, the only thing that we have are people at the medical stores whom we call to check on our orders only

**16:1 I think a lot of it is about appropriate is eeh… the method of using t…… (377:610) - D 16: HH_StM**

I think a lot of it is about appropriate is eeh… the method of using the right medicine and maybe to the right patient and using the right dose, medicines which are still… I mean which are still available and are still new not expired

**16:2 as we use the medicine we have to look at medicine which is about to e…… (645:786) - D 16: HH_StM**

as we use the medicine we have to look at medicine which is about to expire so that we push them forward those that have still time to be used

**16:3 when you give the medication we try to explain to the patient how to t…… (839:1253) - D 16: HH_StM**

when you give the medication we try to explain to the patient how to take it like for example a powder suspension you have to explain to the patient that this syrup is new so you have to use it in a period of seven days or within or otherwise if you use it after its time, the product is less effective so you have to explain to the patient hoping that those people understand so you have to use our mother language

**16:4 just a few ask may just ask for your own information he or she may ask…… (1803:2193) - D 16: HH_StM**

just a few ask may just ask for your own information he or she may ask if I take this medicine will it affect my woman but we know the problem which is why, but if we say we are treating you, we are specifically talking to you and giving you this medication you can’t share it with your woman at home, if she is sick she has to come to the clinic, that purpose of having the two of you safe

**16:5 okay even if she does not feel anything but she has to come for check-…… (2223:2775) - D 16: HH_StM**

okay even if she does not feel anything but she has to come for check-up too as long as they are staying together. We try to have them come at a position of STI, on the other sicknesses we fail, to give the medication to the date or time. We tell them that they should not use it for two days then when you feel well, because of the side affects you stop taking the medication. Because when you come back again we will give you the same medication because you won’t be having the last copy of the prescription sheet so we say use the medicine as we said

**16:6 got Mr Albert, okay I don’t know in the past, but yesterday he was her…… (2917:2987) - D 16: HH_StM**

got Mr Albert, okay I don’t know in the past, but yesterday he was here

**16:7 okay he tries to explain to us how to use a… some of the medication an…… (3510:3740) - D 16: HH_StM**

okay he tries to explain to us how to use a… some of the medication and eeh stock taking rotation of drugs and disposal of expired drugs and even trying to explain as I have said to the patient the use of medication we give to them

**16:8 no sometimes we source it for ourselves (3828:3866) - D 16: HH_StM**

no sometimes we source it for ourselves

**16:9 yes we have actually it in the consultation room. (4160:4208) - D 16: HH_StM**

yes we have actually it in the consultation room.

**16:10 no, I haven’t seen it because usually it stays there at the consultati…… (4749:4825) - D 16: HH_StM**

no, I haven’t seen it because usually it stays there at the consultation room

**16:11 yah it is because as I am saying right now fine maybe they don’t have…… (8579:9011) - D 16: HH_StM**

yah it is because as I am saying right now fine maybe they don’t have at the supplier from outside Swazi Med, or central medical stores but we do experience shortage of drugs okay maybe even delays, we made an order last month, the order has not be delivered we called and they said it’s not ready. We are a short of those drugs and you only find out our problem when it’s time to pick the order and you notice it’s only a few items

**16:12 I don’t remember them calling us and saying you can come and check it’…… (9930:10146) - D 16: HH_StM**

I don’t remember them calling us and saying you can come and check it’s now available, it’s only us who call to check whether anything is there. If you they don’t have any of the drugs then we have to go the other way

**16:13 so we appreciate with your knowledge if you can share with us for some…… (10794:10919) - D 16: HH_StM**

so we appreciate with your knowledge if you can share with us for some time because as you can see we don’t have a pharmacist.

**16:14 yes because at the moment I can’t say we are at the right level,some…… (11000:11227) - D 16: HH_StM**

yes because at the moment I can’t say we are at the right level,some of the things are not in order, so we are trying like what we have been hearing around, we have to get the information on how to go around doing this and that

**17:1 rational use of medicine for me is doing things like ehh… what was agr…… (287:660) - D 17: LU_LubR Hospital**

rational use of medicine for me is doing things like ehh… what was agreed upon by the government that all the facilities should use. They sat down and ehh grouped the medicine and then, they found out that certain drugs can be out there used quite as really needed not a eeh (pause) not every drugs, drugs that would work for certain diseases which are common in the country

**17:2 we are but there is a constraint when, we talk of, sometimes we don’t…… (792:1851) - D 17: LU_LubR Hospital**

we are but there is a constraint when, we talk of, sometimes we don’t get the drugs which were on the list. Sometimes because of the shortage of resources, we tell the patient to go and buy from the pharmacy but as prescribed by the doctor, because they are out there although it is supposed to be here, but because for some time government hasn’t paid the company they don’t deliver as for right now there is a shortage of insulin medicine in the country. It is not that the insulin government is not in the lead, it’s in the lead but they say it’s not in the company where they order from all the necessary drugs that we need. Now the person that is suffering, we do not know whether they buy or not I don’t know. It has been partly I think plus or minus 3months and other specific drugs those out there that people are looking for; they have to be there in the country. If government say they don’t have, what can we do? We just advise the patient to go and buy those who can be able to buy but those who can’t buy we don’t know what they are going to do.

**17:3 some will really do that, they really follow the guideline, most of th…… (2679:3017) - D 17: LU_LubR Hospital**

some will really do that, they really follow the guideline, most of them they even prescribe drugs that are not supposed to be prescribed by the doctors and so when we try to correct them they become furious they call a meeting against us, the pharmacy, that we are discrediting their prescription, yet we are saving the life of a patient.

**17:4 You cannot prescribe for example the medicine, or ehm okay normally th…… (3019:3761) - D 17: LU_LubR Hospital**

You cannot prescribe for example the medicine, or ehm okay normally they do this polypharmacy whereby thirteen or fourteen medications for one patient which is wrong we can do okay if a patient comes with a problem you cannot prescribe pain, say ok there is a patient has flu, you are prescribing panado, you are prescribing ok the mixture of BCO and panado, I understand maybe for the management of the pain, you prescribe all the vitamins multivit, BCO, scopex. Why can’t you take one thing angitsi multivit is mixed, then why do you add vitamin c and BCO again, I understand vitamin c is there in the multivit mix and then you prescribe cold and flu and you prescribe panado, panado and cold and flu, in cold and flu there is panado there.

**17:5 It’s like they really don’t want to be corrected and so they called a…… (3763:4448) - D 17: LU_LubR Hospital**

It’s like they really don’t want to be corrected and so they called a meeting against pharmacy so our challenge is tight, but now we don’t call them now you look at the prescription and you see what you can do. If they prescribe panado 2, 2,2 then you rather reduce to 2 by 1 then cold and flu 1 because now it will make one gram for panado if its 1 not 2,2 but the they don’t separate the anti-inflammatory, they say brufen and diclo are not the same yet it is the same thing and that’s a problem so such prescriptions these are the incidents that you see in the pharmacy of how to do it there. So we must listen to each other in the future so that we are not pointing at each other.

**17:6 a meeting against pharmacy so our challenge is tight, but now we don’t…… (3830:3971) - D 17: LU_LubR Hospital**

a meeting against pharmacy so our challenge is tight, but now we don’t call them now you look at the prescription and you see what you can do

**17:7 So we must listen to each other in the future so that we are not point…… (4361:4448) - D 17: LU_LubR Hospital**

So we must listen to each other in the future so that we are not pointing at each other.

**17:8 it was there but I don’t know what happened but it was introduced and…… (5378:6397) - D 17: LU_LubR Hospital**

it was there but I don’t know what happened but it was introduced and it was going well in 2014- 2015 then there came this thing of committees, there were, okay there were many office committees of which there was only one pharmacist of which she had to go to all these committees now it was an overload to her that all these committees she had to attend cause we have to attend our therapeutic committee when we are here we have to attend everything. In the end, I don’t know even the staff, because we are short staffed vele, nobody has been employed here we are staff from different facilities they are not having anybody though we are three especially pharmacy we are two, then bona manje the prescribers they are about fifteen, fifteen against two, when I am off, fifteen against one. You can see, when can you then attend those workshop or those meetings? You can’t, you rather decline and say ah today I can’t, then it went down down, down. Now I don’t hear of it, although it picked up when I was still there.

**17:10 mmm yah they do at times they do yah sometimes they do but they do it…… (4790:5281) - D 17: LU_LubR Hospital**

mmm yah they do at times they do yah sometimes they do but they do it because of the severity of that disease but they know that if it’s not in the formula, so they can’t prescribe because maybe the patient can’t be treated and this thing of using the antibiotic they told people to refrain from most of the antibiotic so now they prescribe the right popular erythromycin which is not in the government list but we buy from the pharmacy because this patient has been tried all the antibiotic

**17:11 we try but we use every meeting with the government and they tried in…… (6552:7076) - D 17: LU_LubR Hospital**

we try but we use every meeting with the government and they tried in their meetings, nabo matron they tried in their meetings, because another thing, some of the meetings which are supposed to be attended by a pharmacy staff, is attended by the matron or whatever so when they come back, we don’t know what we are supposed to do because now they come with a what do we call it, something like formatting that we are supposed to do this, how, what transpired, who. Why didn’t you invite a pharmacists or pharmacy technician?

**17:12 it was only a suggestion that when we meet or when there is a meeting…… (7321:7454) - D 17: LU_LubR Hospital**

it was only a suggestion that when we meet or when there is a meeting and then we do the training of that one but it’s on the pipeline

**17:13 it will benefit not only the facility even the patients even the nurse…… (7627:7840) - D 17: LU_LubR Hospital**

it will benefit not only the facility even the patients even the nurses will benefit ehh if only we were getting all the drugs that we have ordered, the drugs that are needed by the patient. It will be rational use

**17:14 beside that ehh, besides the training, I would love maybe the training…… (7965:8850) - D 17: LU_LubR Hospital**

beside that ehh, besides the training, I would love maybe the training to be there in those workshops. I don’t know as there is shortage of pharmacists at least training of nurses or reminding them about the rational drug use, I think that could be all or somebody or maybe they undermine the sister because she is too young. Maybe someone from medical stores and they do the training maybe to improve, somebody who would not be from here, somebody from outside. They come and do the training so maybe we can have an intake or a change because people don’t change now, now. It takes time for a person to change. Some they have got a fear of the unknown, some they feel that they know and you would be undermining them, they know better than you, they think that going to workshops in pharmacy they have a degree; you need pharmacist with a pharmacy degree in front then bona they follow

**17:15 uhm on human resources, on HR because I mean as you can see I am about…… (11226:12297) - D 17: LU_LubR Hospital**

uhm on human resources, on HR because I mean as you can see I am about to end, my retirement and now I got a letter to say because what should I say if there is no person to take over so that I train this person. Now I am left with two years, three years it’s no joke and soon I will be leaving, leaving without anybody coming. If there is now somebody coming in this place a new pharmtech all together, we need to show them we don’t do this, we don’t do that, we do this because by order. It should be a younger person so that the younger generation can take this thing from older people especially pharmacy when I go then they bring a new one uyabona and the pharmacist naye also is new, it’s better when you are in-service at least ten years now you can know how to run something, that one there is a gap. There won’t be expectation on what is done on the ground what is being done is not right and they should close the gap they cannot allow the older people to go and retire without knowing a new staff so that these people they train them - at least to the new ones

**17:16 and okay the HR some have already… there are some technicians which we…… (13680:13879) - D 17: LU_LubR Hospital**

and okay the HR some have already… there are some technicians which we were expecting but the problem is the remuneration for pharm techs and the work per annum, work is too much and there is no money

**18:1 maybe every day the word maybe (stammering) aah the rational thing aah…… (300:383) - D 18: LU_Manyv Naz**

maybe every day the word maybe (stammering) aah the rational thing aah I don’t know.

**18:2 yes I would say that, because we are having all the treatment guidelin…… (1065:1296) - D 18: LU_Manyv Naz**

yes I would say that, because we are having all the treatment guidelines for different conditions ehh. Whenever we are stuck we consult the ehh the guideline. You get a clue on how to help that particular client, yah we are, we are.

**18:3 but you only find out that the medication that they suggest or they pr…… (1427:1664) - D 18: LU_Manyv Naz**

but you only find out that the medication that they suggest or they prefer is not in our stock so you find out that you are now handling an alternative which may not be the good one, the preferred medication for that particular condition.

**18:4 okay we usually call the other facilities to find out if they have any…… (2029:2319) - D 18: LU_Manyv Naz**

okay we usually call the other facilities to find out if they have any medication of that sort. Or we have, but it’s not for our ordering books, where maybe it was written by a doctor, maybe we just send them to the clinic with the order, then then the patient comes back with the medication

**18:5 but we order from RFM our medication and sometimes we also order from…… (2684:2757) - D 18: LU_Manyv Naz**

but we order from RFM our medication and sometimes we also order from CMS.

**18:6 okay we usually meet or maybe for common conditions; mmm we usually me…… (4706:4982) - D 18: LU_Manyv Naz**

okay we usually meet or maybe for common conditions; mmm we usually meet maybe once a month or twice depending on the year. If you see someone prescribing maybe something that is different than we used to we correct them and show them on how to prescribe the correct medication

**18:7 I think if maybe our, maybe our medical officers maybe whenever they c…… (5172:5331) - D 18: LU_Manyv Naz**

I think if maybe our, maybe our medical officers maybe whenever they come for their meetings if they, if they maybe just take us through some of the medication

**18:8 not really, not really but we are trying but you will find out that wi…… (6044:6409) - D 18: LU_Manyv Naz**

not really, not really but we are trying but you will find out that with the medication out of stock like right now as we are sitting you find that we just pick them to help the patient but like now we don’t have paediatric formulations for children. We just pick them out on they can be of help to the particular patient but nje it’s a problem for the younger ones

**18:9 all the antibiotics for kids, we order amoxylin, erythromycin, okay we…… (6496:6605) - D 18: LU_Manyv Naz**

all the antibiotics for kids, we order amoxylin, erythromycin, okay we use this smaller one which is for sores

**18:10 what we need from pharmacy maybe it can be whenever they are introduci…… (7412:8056) - D 18: LU_Manyv Naz**

what we need from pharmacy maybe it can be whenever they are introducing new medication it must at least come with a leaflet on how to administer that particular medication so that everyone can be on board on how to use that medication. Because maybe you find that maybe before, they were using DPH maybe they are doing away, to give way, and introducing new medicines you find out that there is no clear steps on how to use that particular medication. Okay maybe if we say for maybe clarity if it is 5L bottles packaged in a box, there is only one leaflet in the box. So when the medicine is supplied, it does not come with the package insert.

**18:11 the problem we are facing is on the always out of stock medication bec…… (8821:9155) - D 18: LU_Manyv Naz**

the problem we are facing is on the always out of stock medication because whenever you want to help that particular client we find that we are just speaking now, and maybe now as we are sitting the particular client is in danger maybe for ehh for other effective medication so that is the problem we facing the issue of out of stocks.

**18:12 yes we do order we always find out that ok for our facility you may or…… (9321:9542) - D 18: LU_Manyv Naz**

yes we do order we always find out that ok for our facility you may order for three months but they just give you what is available maybe it will last two weeks so the other weeks we are out of stock so that’s the problem.

**19:1 I think is the ration per facility or I don’t even have an idea {laugh…… (377:466) - D 19: LU_Nkal**

I think is the ration per facility or I don’t even have an idea {laughs} it is the ration.

**19:2 yes we do practice that one ehh in all most, in all our time with clie…… (1206:1506) - D 19: LU_Nkal**

yes we do practice that one ehh in all most, in all our time with clients we give our client the correct medication at the right time, right doses, we also educate for them to use them correct doses that we have prescribed even when they are at home for the medication that we give them to take home.

**19:3 okay for the client the problem is the language barrier sometimes. The…… (1704:2377) - D 19: LU_Nkal**

okay for the client the problem is the language barrier sometimes. There are sometimes the client don’t get, like, the language that you are using also because we are in the border - some of our clients are from Mozambique, we can’t hear shangaan or portuguese language they are using. So there is a language barrier sometimes also in the grandmothers and others, so when you are writing in the documentation being an envelope, they cannot read clearly what it says, they have to ask the child to help them, you find out that sometimes the grandmother is staying alone and so there is no one to help read the ukuthi what is supposed to be given at what time and what dosage.

**19:4 we have a monthly; we deliberate monthly at the facility in meetings h…… (2713:2884) - D 19: LU_Nkal**

we have a monthly; we deliberate monthly at the facility in meetings how we improve each other’s skills in dispersing the medication and also in prescribing the medication.

**19:5 standard treatment guidelines we have for adults, we started uhm 20mmm…… (3153:3503) - D 19: LU_Nkal**

standard treatment guidelines we have for adults, we started uhm 20mmm I am not sure 2013 about there that they gave us, the regional sister and matron, and they gave us. I think around 2013, they gave every nurse in every ward in the facility. But for children we have the ICI for4.20 we use that one for prescribing not the guideline. We use the ICI

**19:6 okay the method that we are going to use it, whenever if there is a cl…… (3645:4025) - D 19: LU_Nkal**

okay the method that we are going to use it, whenever if there is a client actually they say that it must be where you are prescribing and the category, category umm {laughs} if yah well. They also told us the category that we are in and when we give the medication we are going to look at the category that we are in and not expect other medications that are outside our category.

**19:7 no (5374:5375) - D 19: LU_Nkal**

no

**19:9 okay there are things that we do firstly, we instruct the client pleas…… (5823:6435) - D 19: LU_Nkal**

okay there are things that we do firstly, we instruct the client please go and buy but when we see that the illness is coming more frequent and from others in the community like many are coming requiring this drug, and now then we talk to our sister, sister there is a problem here, we need this drug to have it here so that we can be able to help the clients and stuff like I can make an example for the chronic illness like hypertension and DM, we find that we know that there is a drug that could help the client but we don’t have it. So we talk with sister and then they help us. So some of the drugs we have.

**19:10 aah in service no but we received some sort of training like when we a…… (6824:6954) - D 19: LU_Nkal**

aah in service no but we received some sort of training like when we are going for some other workshop and then it was incorporated

**19:11 like when you go for NCI and INCI and…9.10 then they incorporate it, r…… (6986:7292) - D 19: LU_Nkal**

like when you go for NCI and INCI and…9.10 then they incorporate it, remember whilst giving the drug, it must be at the right time, the right dosage, right patient, so actually there is no a stand-alone, we have never done any stand alone for pharmaceutics, it has just been incorporated in other training.

**19:12 yah its key, it’s needed because it’s one of the essential things, tha…… (7501:8325) - D 19: LU_Nkal**

yah its key, it’s needed because it’s one of the essential things, that you will see when you are giving drugs to clients its key, it stands out either as side effects or it helps the client so it’s very key. Well If I am to look at it the other way, in the clinics it’s the one that is lacking behind ,because we don’t have a pharmacy, anyone in other facility, anyone who happens to come to the prescribing area and find the drugs and gives the pill which is not right when not right because not everyone is trained about how medication is supposed to help because in my facility we prescribe and then the client comes to the dispensing area, it’s not that the nurse ends up from there and comes and disperse, but someone who comes in the dispensary and find the client there so I think somewhere something there is lacking

**19:13 I mean it can be someone who is professionally trained like nurses, we…… (8555:8810) - D 19: LU_Nkal**

I mean it can be someone who is professionally trained like nurses, we do have nurses in the facility but also orderlies, you find that the nurses train the orderly they say aah this is easy we can do like Nazarene, we find that orderly do the prescription

**19:14 we can benefit but once we had, we want to give the best care to our c…… (8965:9297) - D 19: LU_Nkal**

we can benefit but once we had, we want to give the best care to our clients, we want to give extra actually if I can say so we don’t want the client to come back with such a case that you gave me this drug what, what, what. But we want to know that daily we are giving the best so the rational medication scheme could be good for us

**19:15 we need a pharmacist (9369:9388) - D 19: LU_Nkal**

we need a pharmacist

**19:16 okay with the staff that we have could maybe nurses, maybe there can b…… (9694:10408) - D 19: LU_Nkal**

okay with the staff that we have could maybe nurses, maybe there can be a nurse in every facility who is trained with the pharmaceutical who will be assisting on pharmacy department because in some places you see that this drug, how we can use it, there is no one who knows, then we need to call other facilities but if there is a cluster of nurses especially trained. okay we are trying now but there is extra that can be done we need extra, when you come to the working area and start working you will see that we are lacking something unlike when we are in a cluster but when you are actually doing it as a cluster so if a cluster of nurses can be trained with the system that we are doing in the dispensing area

**20:1 to my understanding as long as you say appropriate it is about the rig…… (339:613) - D 20: LU_Siphof**

to my understanding as long as you say appropriate it is about the right patient, right dose, right drugs and appropriate use it goes also with handling, or maybe ordering, storage and ehh stock packing then up to the diagnosis and the supply of the treatment to the patient.

**20:2 now we developed two key projects, the community improvement project s…… (1068:2139) - D 20: LU_Siphof**

now we developed two key projects, the community improvement project so that we know the coordination, the planning, the implementation, the review of everything, so there is good purchasing in the facility, we have our focus person who is in charge of ordering them and receiving them because when the medicine stores delivers somebody must receive according to what we have ordered , if there are reasons that is when the appropriate comes back again because the order is given back to the person and also the packaging , monitoring and supervising the packaging because inappropriate use of drugs can come when the medicines are not… or are just laid anywhere than being monitored up to the patient, the right patient. So there is that too of which depending on the areas of service once in three months the pharmaceutical meeting is held where that thing contributes then makes us aware, it can be once in three months and then we have our resources, the policies, okay we have the doctors’ documents, the nurses list, the medicine list and all the management reports

**20:3 in the facility we decided to draw our own policies downloading from t…… (2178:2916) - D 20: LU_Siphof**

in the facility we decided to draw our own policies downloading from the national medical policies or guideline, in the cardboard you can check there are some books, there is something; in the storeroom the regulation of temperature and monitoring the stockcard when you take something or remove your stuff that you have taken, one tick stands for one thousand units, you record it in that card. Even when the supplies comes in we have got a copy that writes received one thousand of the… one thousand of bottles okay of bottles of panado, I am making an example of panado then in a nutshell we do have our project which is a team holding regular meetings, weekly, regular review, review of the hypothesis for expired drugs and improvement

**20:4 is the nurses from facility but not mentioning names (3065:3116) - D 20: LU_Siphof**

is the nurses from facility but not mentioning names

**20:5 yes, yes, yes because they will know and they learn not anyone run to…… (3299:3629) - D 20: LU_Siphof**

yes, yes, yes because they will know and they learn not anyone run to the storeroom haphazardly, it is then when you know even when ordering what is there in the stock what is not there within the stock then the nursing sister remains the supervisor of everything, she is not allowed to do anything she supervises the whole process

**20:6 I cannot explain much but somehow it is a few group of people from aro…… (3933:4319) - D 20: LU_Siphof**

I cannot explain much but somehow it is a few group of people from around called in a certain meeting or maybe a national meeting for introduction of the guideline, usually if you can’t have the date but there is a big day because I can just make an example, maybe the regional office and a few regional health we say RHMT, health management team not counting the people who are hands on

**20:7 that’s where the gap arises they came to the facility just for the dis…… (4514:5217) - D 20: LU_Siphof**

that’s where the gap arises they came to the facility just for the distribution of the guideline, where you learn on your own, okay the guidelines are very simple then you have to orient yourself on your own and worst part of it, there have been in the past two years they have no… there have been no training from the pharmaceutical, for the medical I cannot know for the medical officers, but for the nurse in charge, there have been a gap so the orientation of the two it has been a gap really without the maybe some promoton then there are improvements - what is the recruitment and the location? You cannot know who is able or specialise on that, be we learn everyday but we are experiencing a gap

**20:8 we do have our pharmaceutical with their cutters and pots, but somebod…… (5583:5697) - D 20: LU_Siphof**

we do have our pharmaceutical with their cutters and pots, but somebody like a health personnel who can look and go

**20:9 but what I can note or request is that if we had a mentor or some ment…… (5826:6069) - D 20: LU_Siphof**

but what I can note or request is that if we had a mentor or some mentors as a region one, its three sub region, north, central and South so that they go with all other mentoring team maybe in a while you…, there is…, they conduct a few studies

**20:10 or because we also lack some pharm technicians we have some nurses who…… (6100:6455) - D 20: LU_Siphof**

or because we also lack some pharm technicians we have some nurses who are also, who are applying the medicines to the patients who are doing all these things since there is no pharmtech, if really there can be a mentor, if there could be a mentor so that those around can maybe change, also be mentored even the writing then the documentation and the rest

**20:11 thank you, uhm there are issues of the gap really (chuckles), there ar…… (7077:7238) - D 20: LU_Siphof**

thank you, uhm there are issues of the gap really (chuckles), there are also stock outs maybe due to lack of knowledge of what is in the central medicine stores.

**20:12 Then we keep on ordering, then I think it’s also lack of communication…… (7239:7841) - D 20: LU_Siphof**

Then we keep on ordering, then I think it’s also lack of communication, along the lines there is a format, a gap then we can experience some out of stock which is not supposed to be an out of stock then maybe the medicines will be out of stock just in the medical stores, yes in the medical stores when they were hit hard by the sanctions from medical stores I don’t know maybe the RFM team - and went back from that one which was not communicated to be implemented as a local so we were out of stock for some months from the medical stores, so I think those are the out of stock I can be talking about.

**20:13 Sometimes the medicines they come towards expiry date when there is su…… (7843:8146) - D 20: LU_Siphof**

Sometimes the medicines they come towards expiry date when there is supposed to be ample time before the expiry date. Today its August maybe in October or the following month, September, and this is September, so the drugs they are pushed so that the responsibility (chuckles) is pushed to someone else.

**20:14 Sometimes the medicines they come towards expiry date when there is su…… (7843:8273) - D 20: LU_Siphof**

Sometimes the medicines they come towards expiry date when there is supposed to be ample time before the expiry date. Today its August maybe in October or the following month, September, and this is September, so the drugs they are pushed so that the responsibility (chuckles) is pushed to someone else. Actually this is when we find expiries that these days are otherwise as a facility we are a high volume, we are a high volume.

**20:15 Then maybe some other factors contributing to the out of stock with fa…… (8274:8493) - D 20: LU_Siphof**

Then maybe some other factors contributing to the out of stock with facilities is small it has become small, maybe it was okay initially in those years when I was not born, it is still the same property since I came here

**20:16 it was like this but this facility is high volume and high volume pati…… (8526:9148) - D 20: LU_Siphof**

it was like this but this facility is high volume and high volume patients of all sorts of diseases so even our medicine storeroom they are very, very small, they can’t contain what we wish we can order so then the out of stock can happen when that quantity is finished because we didn’t have anywhere to keep our stock, so it’s just a burden or a list of challenges, but not forgetting it also comes back to what you were saying earlier what I can…, which I can say other issues okay you must mention the small storeroom and the out of stock maybe the performance level in the high volume facility because pharmtechs then

**20:17 training, can I add the training. The training like all other programm…… (9151:9440) - D 20: LU_Siphof**

training, can I add the training. The training like all other programmes then the review of medicine list in line with the essential medicines package, yes because nurses all maybe work from latest in some work, or their work is dated some years back in this time when there is so much need

**20:18 so really the essential medicine package has to be reviewed and the me…… (9443:10464) - D 20: LU_Siphof**

so really the essential medicine package has to be reviewed and the medicines have to be decentralised from the health facilities to the public health, public health and the whole clinic that we are any clinic cause even for the maternity for pregnant woman which is under… according to the medicine list it is not supplied to clinics it stands out there in the hospitals there is a huge gap rising in the accessibility, in the affordability and availability. when you take care of a stranger two months and, I am just making an example of some of the list which has to be decentralised today to the clinics to everyone in fact on care then they tell you “ no go to the hospital or go to typical Sitobela”, and they don’t have money and they will not go there, to look after prevention of eclampsia I mean prevention of pre-eclampsia well the person you may never know what happens and think otherwise and not come back to Siphof because there is no, because the patient has not gone there to the hospital to see…

**20:19 I can also add that they can facilitate the clinics or we request that…… (11081:12045) - D 20: LU_Siphof**

I can also add that they can facilitate the clinics or we request that when they say appropriate they must supply or request that if they strengthen the human resource by adding medical officers who have to be full time okay as for here we have our TB medical officers who come for outreach once a week so everything because quality management requires most of us to work together to be but even for the hypertension we don’t know if they have been receiving medicine for a year or half a year and then for a year we need maybe to have an intervention for that and also monitor the level of improvement, actually we can keep doing like that for some time - we cannot check if the BP is clear - it has become stabilised. It has become inappropriate when that patient is not reviewed or maybe the review is done inappropriately in that improvement, in that level of improvement so it requires the medical organisation of the medical officers to the health facility

**20:20 maybe I can also add that they can facilitate the clinics or we reques…… (11075:12045) - D 20: LU_Siphof**

maybe I can also add that they can facilitate the clinics or we request that when they say appropriate they must supply or request that if they strengthen the human resource by adding medical officers who have to be full time okay as for here we have our TB medical officers who come for outreach once a week so everything because quality management requires most of us to work together to be but even for the hypertension we don’t know if they have been receiving medicine for a year or half a year and then for a year we need maybe to have an intervention for that and also monitor the level of improvement, actually we can keep doing like that for some time - we cannot check if the BP is clear - it has become stabilised. It has become inappropriate when that patient is not reviewed or maybe the review is done inappropriately in that improvement, in that level of improvement so it requires the medical organisation of the medical officers to the health facility

**21:1 would say it’s the appropriate use of medicine in terms of prescribing…… (431:917) - D 21: LU_sith**

would say it’s the appropriate use of medicine in terms of prescribing the proper medication and the proper dose taking into account the patient particularity because all the patients are not the same and also the kind to be somehow of , as an SMO I don’t think other people might actually be interested in that trying to be also effective with your own organisation not to waste, not to overprescribe but also not to underprescribe because all you want is to save drugs, so that is it.

**21:2 I would say yes because we are not too many prescribers, yes we are ju…… (1033:1324) - D 21: LU_sith**

I would say yes because we are not too many prescribers, yes we are just three doctors and some few nurses. As doctors we have meetings where we sit down and discuss how we prescribe, how the prescriptions are being drawn, we discuss about it and so far I could say we are using as commended

**21:3 but then on the nurse side I wouldn’t take any responsibility to talk…… (1325:1668) - D 21: LU_sith**

but then on the nurse side I wouldn’t take any responsibility to talk on the fact but I want to believe that even the meeting and discussions we make and all the precautions we take in our meeting and all those things I want to believe that even the nurse they prescribe based on the guideline cause we really insist on following the guideline

**21:4 besides the guideline we have meetings where we discuss about the drug…… (3354:4142) - D 21: LU_sith**

besides the guideline we have meetings where we discuss about the drug uses, prescription pattern and then we discuss about other things. But for now the meetings are based on doctors’ side we share the information, we also have the pharmaceutical therapeutical committees where we discuss about those things but there also we still need to revise the meetings because we don’t meet as usually like we should. It is there where we discuss about drugs, we discuss about the side effects, drug shortages, prescription habits, new drugs that are around, identity drugs when we are short of some given drugs then in the meetings we discuss since we can don’t have these drugs we can be using these drugs for this day because it can still help on that condition so that’s the intervention we do

**21:5 a new one actually here we didn’t do it but we have meetings with the…… (4431:4804) - D 21: LU_sith**

a new one actually here we didn’t do it but we have meetings with the ministry of health every month called the breakfast meeting in one of those meetings, I remember once we had a presentation and we actually asked for some drugs that are available in bigger hospitals to be also made available in health centres to help doctors to reduce what we call unnecessary rationing

**21:6 I want to believe that the ministry of health has got the presentation…… (4912:6291) - D 21: LU_sith**

I want to believe that the ministry of health has got the presentation on power point made by Sith in which they are asking the ministry to revise some part that are in the guideline to make more drugs available for hospitals, we know that in Swaziland there are drugs that you can’t find in clinics. There are drugs that you can’t find in health centre though those drugs are available in the country but you find out that it’s only a special quantity. There are even some drugs that are not even there, if a patient actually needs them, we recommend and ask that they order it especially for the patient for a given date. So these are the kind of issues that happen but within the facility if it happens I will take one case for example like with CPX at each level we would travel to the Dvk you will go with CPX, while actually working in the clinic, now you need CPX that’s how I once learnt that it’s not that in Swaziland there is no IV CTX but it’s not allowed to exit the health centre. In such a case we will end up, I mean end up referring it while actually we know how to treat it. So we are referring that patient not just because we are incompetent but because there are drugs that are not available at your level. So the patient should go to a level where the drug is available but we haven’t taken it as an issue in our meetings we just follow the flow

**21:7 it happens a lot sometimes it even happens that you are given a situat…… (6495:7323) - D 21: LU_sith**

it happens a lot sometimes it even happens that you are given a situation that you prescribe a drug you wouldn’t normally prescribe for that patient but because that stock of antibiotic for example… It happens that the only antibiotic you have maybe is benzyl amoxyl then, so when a patient come in with whatever condition she or she might have, I have no choice but to prescribe the only available antibiotic everyday but you know that you needed to give the patient a different one but because you can’t tell the patient go home there are no drugs, a patient who has already paid, he has been through the line. He has come to you for prescription so you prescribe but you know that there is little that the patient will benefit from this but just take this for now and come back and it happens more than once it really happens

**21:8 it happens a lot sometimes it even happens that you are given a situat…… (6496:7323) - D 21: LU_sith**

it happens a lot sometimes it even happens that you are given a situation that you prescribe a drug you wouldn’t normally prescribe for that patient but because that stock of antibiotic for example… It happens that the only antibiotic you have maybe is benzyl amoxyl then, so when a patient come in with whatever condition she or she might have, I have no choice but to prescribe the only available antibiotic everyday but you know that you needed to give the patient a different one but because you can’t tell the patient go home there are no drugs, a patient who has already paid, he has been through the line. He has come to you for prescription so you prescribe but you know that there is little that the patient will benefit from this but just take this for now and come back and it happens more than once it really happens

**21:9 I don’t know how much of help you are going to give me, but I think re…… (7521:8060) - D 21: LU_sith**

I don’t know how much of help you are going to give me, but I think really that there are some drugs that are there in hospitals that need to be brought to the health centres as well, there are a lot and while we look at the onset of health workers you find that actually the difference between me and these doctors in bigger hospital is nothing, so if you refer me, move me to a bigger hospital then I get access to those drugs, I would prescribe them now that I am here you say no I can’t access those drugs I don’t know, it’s not fair,

**21:10 like before I became SMO here the one who was here went to Mbabane gov…… (8090:8354) - D 21: LU_sith**

like before I became SMO here the one who was here went to Mbabane government hospital. So if those doctors couldn’t prescribe those drugs here, it’s now a big question is what new qualification has he got that has given in rights now to have access to those drugs?

**21:11 it’s still the very same professional that we see. So I don’t really u…… (8384:9464) - D 21: LU_sith**

it’s still the very same professional that we see. So I don’t really understand whats the point of transferring things there and not this side while actually the competencies of that doctor don’t differ, because I would understand maybe if it is for a specialist only but not a general practitioner like myself so it doesn’t really make sense to me and we really need those drugs. Sometime they still give us but when you look at the amount you are given its really not encouraging because, if you prescribe to two or three patients then it is finished and these sicknesses they also come very frequently so you end up not treating all the patients. They assume you don’t have too many patients suffering of that sickness and they just give us a little while in big hospitals they are just pumping them so the government must do a research on which cases are around Sith. I remember we had a patient who came here and we started treating her but before the treatment for three days or four days then we ran out of stock and then we had to refer the patient to another facility

**21:12 yes they need to revise actually the guideline and then the distributi…… (9649:10813) - D 21: LU_sith**

yes they need to revise actually the guideline and then the distribution of medicine, they need to revise which one should be in hospitals and which one should be at health centres and actually to review it unless they should also feel that those patients should be referred and then they should also solve the issue pleasantly because there are serious transport issues and the condition of the road, they should actually make Sith a special place, there are some places that they need to take into account and not a treat them like just any other health centres. Sith needs to be given the same capabilities as those hospitals, you can’t say that they are at the same level yet they don’t have the same capabilities. Sith needs the highest capabilities because there are other or that actually my view and in fact they need to make sure that Sith is able to handle most of the patients inside rather than referring them, because of these struggles, even the transport issue, the road issue and even if they give us trucks today after a year they will be broken down because of the road and then they would be sitting there for months and years

**21:13 think that’s the main issue and by saying that we can see that it’s no…… (11127:11387) - D 21: LU_sith**

think that’s the main issue and by saying that we can see that it’s not just the drugs but also other things like the blood transfusion, the theatre and all those things should also be included in Sith such that it does not need to always refer to outside

**22:1 I personally think that the appropriate use of medicine is when the he…… (350:840) - D 22: LU_StP**

I personally think that the appropriate use of medicine is when the health care professionals are prescribing or giving out the medication that is guided by the essential medicines list, they are giving out the medication that is recommended within their clinical list or within their class list that is not outside no I am sorry that is within the class list and even though on exceptional cases some medication that is outside the class list especially for the certain classes can be used

**22:2 definitely we are, as a facility we have been given by… we have been g…… (948:1142) - D 22: LU_StP**

definitely we are, as a facility we have been given by… we have been given by the ministry, some guideline that we are using, the essential medicine list and we are still using the latest edition

**22:3 we requested, we already were having the guideline because we were req…… (1897:2465) - D 22: LU_StP**

we requested, we already were having the guideline because we were requesting you know from our regional matron and sisters who were supervising us, or who were frequently visiting the St Philips clinic to say here we are an HIV and TB facility at least we need to be aware of what is happening as far as the prescribing in the clinic facility is concerned. So that is when we got to be given the essential medicines list of course that time we didn’t have many of them I am sure it was between the managers who were having it and we were sharing it amongst the staff

**22:4 so they were just given to us we never attended or we never received t…… (2742:3877) - D 22: LU_StP**

so they were just given to us we never attended or we never received training of some sort that is guiding us on the essential medicine list and then at the facility because we really thought that there was a need for us you know to be aware of what are the standards that is required of us, we were inviting this lady I can’t remember her name but she was coming to us through ICAP who was supporting us, so she was basically focusing on the pharmacy and she was engaging us on these training especially for the nurses whereby in that time we have stations where we would sit down and discuss the medication side effects, look into the essential medicine list and she would also sit with us to discuss how we are supposed to be prescribing the medication if we are prescribing what is recommended within the essential medicine list or something that is outside the list and again we are also looking at the issues of poly pharmacy. I am sure that was then around 2014, 2015 then afterwards we had this lady from ICAP and again ICAP was also about to move in the region so since then we never had these training again following that.

**22:5 we also rely on these standard operative procedures that we get from t…… (4038:4457) - D 22: LU_StP**

we also rely on these standard operative procedures that we get from the ministry and also the relevant guidelines for the particular maybe condition that we have to manage for example on TB there are guidelines for TB, mother and child welfare services there is again materials that is relevant to that again. So we rely basically on the standard guidelines that are provided for us outside the essential medicine list

**22:6 I forgot to tell you we are a non-profit making organisation, Non-Gove…… (4622:5358) - D 22: LU_StP**

I forgot to tell you we are a non-profit making organisation, Non-Governmental Organisation so ever since we took over the facility or even before when we were running an HIV and TB facility we were sourcing out medication for non-communicable conditions on our own so it’s only the anti and TB medications that we have been getting through the central medical stores so we do run at challenges because if you have seen the medication we have been using and the course of the medication we have been using, anything for us since we are a non-profit organisation we are really challenged because we have to buy this medication for communicable conditions and the treatment vele is so hard for us on our budget to follow on our guidelines

**22:7 First, I would say as a facility we have been trying to get few or to…… (5505:6257) - D 22: LU_StP**

First, I would say as a facility we have been trying to get few or to order our medication through the central medicine stores and this has been our greatest challenge and we have been pushing for almost two years now and we are still unable to get the medication. So I think if we could be able to get some of these medications through the central medical stores, I am sure it can actually also bring down the organisational budget considering that we are a non-profit making organisation at the same time I think it would be cheaper maybe the people at the central medical stores could also dedicate themselves time to pay a visit to the facility see how the pharmacies in the facilities are being run and then they can also engage us on the training.

**22:8 I think it would be cheaper maybe the people at the central medical st…… (6011:6257) - D 22: LU_StP**

I think it would be cheaper maybe the people at the central medical stores could also dedicate themselves time to pay a visit to the facility see how the pharmacies in the facilities are being run and then they can also engage us on the training.

**22:9 Yes if I can be honest I feel as an organisation we tried to set up th…… (6259:6808) - D 22: LU_StP**

Yes if I can be honest I feel as an organisation we tried to set up the pharmacy on our own but you may find that maybe there is a standard in which we are required to set the pharmacy you know, purchase the medication within the shelf but we have no such information and maybe you may find that maybe the people at the central medical stores they are too busy to come down to the facility for such things we need training even on the stock out, how to update them and document on them. They never give such training, so whenever we go for workshops.

**22:10 Of course they do unveil themselves but I think the time is just too l…… (6810:7876) - D 22: LU_StP**

Of course they do unveil themselves but I think the time is just too limited for us to do all this learning on what is actually happening on the ground and I think it would also be good for people to actually visit these grounds because we are talking about real life situations, we are talking about drug stock out and what have you. They can really see what has been happening on the ground rather than when we are talking sitting in the workshops which is not enough for me even on the use of essential medicine. Whenever, let’s say maybe whenever there is a new guideline that has come up in clinics it would be worthwhile for those people let’s say for instance those of the TB programme if they do make an effort where they follow up even in the facilities to see if really we are now aware of the medication and the results and whatever, so again even for the essential medicine list if we could also be followed right in the facilities, be mentored on how it is supposed to be used and also they keep refreshing us to make sure we really follow it to the end.

**22:11 I am also concerned, you know for me it seems like the focus for the m…… (8718:9815) - D 22: LU_StP**

I am also concerned, you know for me it seems like the focus for the medication, for the management and what have you, the focus has moved especially to look at HIV and TB and I am wondering how much effort is being done for these other non-communicable conditions because as an non-governmental organisation in this community I have also realised that we are really having huge numbers of people, an influx of people coming from other facilities to access the service especially for their non-communicable conditions. Is there a way that the government could also do maybe to ensure that even the facilities other than my own facility we are stocked well for these non-communicable conditions. And again even the training I am seeing that the focus is just on HIV and TB, for me HIV is like you know, just like any other condition it is now like a chronic disease it is now manageable, it’s no longer a pandemic condition like diabetes and hypertension. HIV is also new a chronic condition and we also need to do as much training as we were doing for HIV for even these non-communicable conditions

**22:12 again I am also concerned, you know for me it seems like the focus for…… (8712:9816) - D 22: LU_StP**

again I am also concerned, you know for me it seems like the focus for the medication, for the management and what have you, the focus has moved especially to look at HIV and TB and I am wondering how much effort is being done for these other non-communicable conditions because as an non-governmental organisation in this community I have also realised that we are really having huge numbers of people, an influx of people coming from other facilities to access the service especially for their non-communicable conditions. Is there a way that the government could also do maybe to ensure that even the facilities other than my own facility we are stocked well for these non-communicable conditions. And again even the training I am seeing that the focus is just on HIV and TB, for me HIV is like you know, just like any other condition it is now like a chronic disease it is now manageable, it’s no longer a pandemic condition like diabetes and hypertension. HIV is also new a chronic condition and we also need to do as much training as we were doing for HIV for even these non-communicable conditions.

**22:13 thank you so much I am also hoping so because right now as you can see…… (10813:11090) - D 22: LU_StP**

thank you so much I am also hoping so because right now as you can see there is the essential medicine list guideline which is a sample. It’s a 2012 one, but if we can look at the guideline for HIV almost every two years there is a new edition but we are holding on to this one.

**23:1 it’s to give medicine that will actually help the client, in terms of…… (478:591) - D 23: MA_Can**

it’s to give medicine that will actually help the client, in terms of the drugs, the time presenting the complaint

**23:2 think we are practicing rationally because in the first place we are u…… (756:967) - D 23: MA_Can**

think we are practicing rationally because in the first place we are using the standard treatment guideline to refer on what medicines to actually give in the particular condition that the patient might be having

**23:3 I would actually say that every Friday we mostly have our inservice me…… (1641:1945) - D 23: MA_Can**

I would actually say that every Friday we mostly have our inservice meetings where we just eeh, discuss or maybe we present, maybe nurses also have something that they want to ask or any grievances concerning the medications and the conditions we are seeing in the facility and we try and help each other

**23:4 I think we need one, let me say we are not fine just one week especial…… (2608:2702) - D 23: MA_Can**

I think we need one, let me say we are not fine just one week especially on the pharmacy point.

**23:5 it’s not a big problem, its naturally actually when they are ordering…… (3290:3600) - D 23: MA_Can**

it’s not a big problem, its naturally actually when they are ordering our medicines we have a problem of half of the order being delivered and not having the all the medication delivered and also I would want to highlight that at the end of it all they do deliver some of the medicines but not at the right time

**23:6 I think nothing except that we are really having a big problem stock o…… (4124:4517) - D 23: MA_Can**

I think nothing except that we are really having a big problem stock out where the orders from the central medical store are not delivered at all or in time and the whole incident sometimes the central medical stores they deliver much more than we order and sometimes its things that we don’t even require and now we have to find a way to try and return them or share with other facilities, yah

**24:1 I think it’s having enough stock and prescribing the right medication…… (393:481) - D 24: MA_Geb clinic**

I think it’s having enough stock and prescribing the right medication to the right client

**24:2 maybe sometimes, okay challenges like maybe we don’t have the medicati…… (836:1134) - D 24: MA_Geb clinic**

maybe sometimes, okay challenges like maybe we don’t have the medication we want at that time when we want to give it to the client at that time, or sometimes maybe not sure about the conditions, some other conditions are really difficult you don’t know about what to use on the other hand you refer

**24:3 okay usually we have challenges maybe two or three weeks before the me…… (1832:2176) - D 24: MA_Geb clinic**

okay usually we have challenges maybe two or three weeks before the medicines expires we have some form for returning it, so far we haven’t but we have the form, it has the expiry date number the medication that is expiring, but usually you find that on the other end they give us medication which is about to expire, so it’s a list of expiries.

**24:4 the ones though the ones which are about to expire, we had the convers…… (2654:2859) - D 24: MA_Geb clinic**

the ones though the ones which are about to expire, we had the conversation, we had last week but nje we used to record that there is stock which is about to expire but I have never seen them taking it back

**24:5 we need some medication, some medication it’s an on and off thing but…… (5309:5490) - D 24: MA_Geb clinic**

we need some medication, some medication it’s an on and off thing but some have been gone for a long time, like antiseptic for the cleaning wounds and something to apply to the wound

**24:6 just the shortage of medicines even for conditions like diabetics and…… (5989:6107) - D 24: MA_Geb clinic**

just the shortage of medicines even for conditions like diabetics and hypertension, so we end up referring all the time

**24:7 very far so it’s difficult okay, so the patients are even complaining…… (6218:6503) - D 24: MA_Geb clinic**

very far so it’s difficult okay, so the patients are even complaining that they have to go to Mank every time so they end up discontinuing their medication, so how will they heal if they are not taking their medication but they have no money to go to the hospitals or buy the drugs

**24:8 okay okunye maybe to do with appropriate use of drugs it’s the orderin…… (6780:7061) - D 24: MA_Geb clinic**

okay okunye maybe to do with appropriate use of drugs it’s the ordering, because at times you find us having a lot we are not using. Sometimes we order more than we need so maybe if they can help us, or encourage us on the issue of stock taking and ordering that could help us a lot

**25:1 RMU is about prescribing the correct medicines for patient illness and…… (904:1165) - D 25: MA_TLkc**

RMU is about prescribing the correct medicines for patient illness and ensuring that it fits the motve of care for the patient. Rational use is different in cases where processes like screening are involved or if there is long-term use of a particular medicine.

**25:2 What is outstanding for us a facility, which I believe is unique, is t…… (1261:1481) - D 25: MA_TLkc**

What is outstanding for us a facility, which I believe is unique, is that we do our medicine labels in SiSwati. The labels have reasons why the patient is taking the medicine and directions on how to take these medicines.

**25:3 We do our work as a team and that ensures that there are lots of quali…… (1594:1927) - D 25: MA_TLkc**

We do our work as a team and that ensures that there are lots of quality assurance processes i.e. more than one person is able to review prescribing and dispensing practices. Also, the dispensary is in the same room as the doctor’s room. This means that anytime the dispenser picks an error, they immediately interact with the doctor.

**25:4 We also have prescribing guidelines that our physicians who come from…… (1931:2179) - D 25: MA_TLkc**

We also have prescribing guidelines that our physicians who come from Southern Africa and America use. We have the national guidelines and also use an electronic database that has a reference list. We have the SD STG/EML but we’re not using it much.

**25:5 Also, we have a senior pharmacy committee that comprises of the physic…… (2183:2589) - D 25: MA_TLkc**

Also, we have a senior pharmacy committee that comprises of the physicians and nurses (we do not have pharmacy personnel). This committee discusses stock issues and prescribing guidelines. We have also used this committee to motvate for new drugs that we could not order from CMS to be added onto our list. The process takes a long time, hence we find ourselves procuring outside CMS for urgent conditions.

**25:6 We have a lot of internal trainings. We have challenges with external…… (2677:3046) - D 25: MA_TLkc**

We have a lot of internal trainings. We have challenges with external trainings due to the scheduling of going out for the outreach clinics. So you find that we get invited to certain trainings but our core aim is to help communities; and if that training is happening at a time when we have to be on outreach we tend to struggle to find someone to send to the training.

**25:7 Yes we have a number of challenges. Stock-outs are a big challenge bec…… (3151:4006) - D 25: MA_TLkc**

Yes we have a number of challenges. Stock-outs are a big challenge because we run out of stock due to CMS not supplying the quantities that we order. Another challenge is knowing if RMU is practiced by the patients themselves. Adherence is a big challenge as shown by other programs in the country such as HIV/AIDS and TB. As much as we make an effort to give the right medicine to the patient, it is difficult to know whether the patients are using them correctly. Diagnosis is another challenge, as we do not have diagnostic processes in place as a country. We therefore work with differential diagnoses and this makes it difficult to adhere to guidelines. We end up practicing the best medicine we know. Swaziland is not peculiar in terms of not having diagnostic processes. Generally it is not financially wise to invest in a lot of diagnostic testing.

**25:8 Yes we have a number of challenges. Stock-outs are a big challenge bec…… (3151:4008) - D 25: MA_TLkc**

Yes we have a number of challenges. Stock-outs are a big challenge because we run out of stock due to CMS not supplying the quantities that we order. Another challenge is knowing if RMU is practiced by the patients themselves. Adherence is a big challenge as shown by other programs in the country such as HIV/AIDS and TB. As much as we make an effort to give the right medicine to the patient, it is difficult to know whether the patients are using them correctly. Diagnosis is another challenge, as we do not have diagnostic processes in place as a country. We therefore work with differential diagnoses and this makes it difficult to adhere to guidelines. We end up practicing the best medicine we know. Swaziland is not peculiar in terms of not having diagnostic processes. Generally it is not financially wise to invest in a lot of diagnostic testing.

**26:1 like avoiding under treatment of patients and over treatment of patien…… (554:625) - D 26: MA_Maft**

like avoiding under treatment of patients and over treatment of patients

**26:2 yes we are trying but sometimes we have some challenges, if maybe a ne…… (729:1054) - D 26: MA_Maft**

yes we are trying but sometimes we have some challenges, if maybe a new staff nurse has come to the clinic or like maybe from school because sometimes she can underdo it or overdo it but like orientation, if we do orientation, if we do proper orientation and if that person keep on practicing so that person usually avoid that

**26:3 okay we have this tool that we are using. It’s standard treatment plan…… (1173:1279) - D 26: MA_Maft**

okay we have this tool that we are using. It’s standard treatment plan that we have, so we usually use it.

**26:4 everything is there, although some of the things that are there we are…… (3444:3772) - D 26: MA_Maft**

everything is there, although some of the things that are there we are not supposed to like eeh okay at RFM clinics we have a guide to order drugs. You don’t order everything that is not in the guide like let me say usually they have diclofenac; there is the injections and tablet, the injection you can order but the tablets no.

**26:5 no they are not (pause) it is expensive for at least everyone because…… (4588:4850) - D 26: MA_Maft**

no they are not (pause) it is expensive for at least everyone because we even have to write a prescription of everything, because like ehm I think last month we even had a stock out for even panado, so we try to ask from other clinics then we were given something

**26:6 I think training whether it’s in service training or something but we…… (6512:6696) - D 26: MA_Maft**

I think training whether it’s in service training or something but we need training on the rational use of medicines maybe twice a year because there is staff rotation in every 6months.

**26:7 like nje in family planning we only have injections, we don’t have ora…… (7281:7381) - D 26: MA_Maft**

like nje in family planning we only have injections, we don’t have oral. But it’s just for mainly nje

**26:8 because angazi maybe if there are senior people, if they can maybe vis…… (9196:9468) - D 26: MA_Maft**

because angazi maybe if there are senior people, if they can maybe visit the clinics maybe twice a year or maybe every good times because it’s what you find sometimes. You find that some clinic A is highly overstocking nje, in their pharmacy yet then clinic B has nothing,

**27:1 for them for instance if maybe they have come to the clinic especially…… (827:1288) - D 27: MA_Mahl**

for them for instance if maybe they have come to the clinic especially the older patients, the grannies, they will feel maybe if you prescribe something for them for example someone suffering from flu obviously we would give them cold and flu, there is panado…, there is paracetamol the but most of them would say I want panado alone that’s what I need meaning if you give them cold and flu they would say you didn’t give them the rightful medicine for them, yes

**27:2 yes I would say we are practicing, for instance usually if I am coughi…… (1403:2049) - D 27: MA_Mahl**

yes I would say we are practicing, for instance usually if I am coughing today and have a cold, I would give you cold and flu to reserve the paracetamol if I give them additional paracetamol, it would be an overdose to you which is not a good thing. I think that the problem when dispersing medication are you trying to explain to the client, this one is going to cover you on this and this and that because at the end of the day you want the client to be satisfied when they leave the facility and happy knowing that whenever they are taking the medication, this is what they wanted, without complaining about the medication, they are taking home

**27:3 yes we are using it but nje, when you are coming to the issue of the s…… (2984:3371) - D 27: MA_Mahl**

yes we are using it but nje, when you are coming to the issue of the supply of medicine. In the book you may find maybe for instance way back I don’t know there was a new order form may be the new order form has just been revised, it has the new medication that has not been allowed to be ordered by the clinics, so we can’t get some of the medication that is recommended in the guideline

**27:4 In a way I think the standard treatment guideline needs to be revised…… (3681:4048) - D 27: MA_Mahl**

In a way I think the standard treatment guideline needs to be revised at least something is needs to be done currently. The new order form has to be revised also which will probably answer a lot of things in terms of why you are talking as if somebody is everyday it one of those drugs you are not allowed to order it, in other way there is no way to justify the drugs

**27:5 okay I think it’s with the central medical stores cause we order every…… (5558:5941) - D 27: MA_Mahl**

okay I think it’s with the central medical stores cause we order every month or the other thing that could be a problem is that if I order something, they give me what is ordered but not in that order. Okay you find that Canlisation at current we don’t have Canlisation there is nothing informing us ukuthi is it a human error or the drug is out of stock, I think that’s the problem

**27:6 o not having enough stock, say no stock plus on top of that this one e…… (6749:7947) - D 27: MA_Mahl**

o not having enough stock, say no stock plus on top of that this one expires at the end of the month, like they are pushing the problem to us, we tell them that no we are not receiving this one put it aside then the following day the manager called and said supplies are being supplied this and that but we did not corporate this drug according to our order we did not order this drug and they say no we going to send a car to pick it the truck come again in a space of two weeks the unfortunate part for them, the truck will arrive around four after lunch to say the is going back if supply with something we order, how could you supply something I didn’t order, how do you expect me to accept something I did not order, if I take it I am acknowledging that I ordered a drug, so I am saying that I am having cartulary so get to the RFM and before it was Friday they neither called on Monday complaining that we return drugs without maybe any communication they expected me to hold on to the drug, it was wrong for me, yet they sent something I did not order; it was wrong if I could just order they want to say we keep drugs, drugs are expiring in the clinics other than being given to the clients.

**28:1 I think it depends with the prescribers, sometimes you find that its n…… (703:1093) - D 28: MA_Mank**

I think it depends with the prescribers, sometimes you find that its not rational especially vele sometimes they prescribe rations they prescribe every symptom for every patient even if you were here last week, you come back still they give you the some medication, I don’t think they check before prescribing, or do a follow up of the last time you took and we ended up calling her (laughs)

**28:2 oh we do call the doctor sometimes to find out why, so nje i-coordinat…… (1222:1546) - D 28: MA_Mank**

oh we do call the doctor sometimes to find out why, so nje i-coordination sometimes and yah they give you the that then we ended up deciding to give a note to the doctor because there are those Cubans ones, they say I want that give that person that and you will be seeing that this is not right, they ended up not giving up.

**28:3 because sometimes you find out that there are some medicines that are…… (3152:3435) - D 28: MA_Mank**

because sometimes you find out that there are some medicines that are not even supposed to be prescribed by the nurses, and you find the nurse changing the prescription from the to the and its not supposed to happen like that, its supposed to be the doctor who is responsible for that

**28:4 yes the drug shortage is the most serious one and the other thing I th…… (4616:4948) - D 28: MA_Mank**

yes the drug shortage is the most serious one and the other thing I think that its to me is not prescribing anyhow, yes and the nurses I think they should be told, I don’t know because they are supposed to be taught at school, they know this is the as a nurse I amnot supposed to prescribe that drug I think maybe that is the doctors

**30:1 it’s using amount of drugs according to, oh sorry (laughs) it is using…… (406:580) - D 30: MA_Mkhul**

it’s using amount of drugs according to, oh sorry (laughs) it is using the amount of drugs needed for the, corresponding to the number of patients a day per day and per month.

**30:2 in the training they cannot, the inservice we have requested inservice…… (6145:6282) - D 30: MA_Mkhul**

in the training they cannot, the inservice we have requested inservices from the regional pharmacist but since today nothing has happened

**30:3 mmm (laughs) at the moment it’s a disaster we don’t have medication, w…… (6963:7557) - D 30: MA_Mkhul**

mmm (laughs) at the moment it’s a disaster we don’t have medication, we have run out of every drug since, it’s been almost two years something like that some drugs have been out of stock so it’s a disaster. Some drugs have been out of stock so we are having difficulty there, even the patients are now blaming us, having it’s like the drugs are, it’s us who are buying the drugs, they don’t understand that there is a process, the government process that is delaying the supply of drugs they don’t understanding because they just think it’s us the nurses who don’t want to give them their drugs.

**30:4 mmm, they don’t, the STI is a disaster because there is no drugs becau…… (7773:8088) - D 30: MA_Mkhul**

mmm, they don’t, the STI is a disaster because there is no drugs because we know how critical it is and their drugs it’s been a year out of stock with hypertensive it’s been two years out of stock they have been buying the gogos’ They are no longer coming; they just go and buy those who can’t afford carry their ids

**31:1 I think appropriate use of medicine is (pause) the (pause) giving the…… (331:699) - D 31: MA_zond**

I think appropriate use of medicine is (pause) the (pause) giving the patient or the client the best medicine that after checking all the history and then the investigation before giving that medicine that is going to help that the client. You… and for other conditions, for conditions that are… difficult having a guideline you try to follow the guideline that is that

**31:2 yes I think we are practicing it but at times you will find that other…… (813:1212) - D 31: MA_zond**

yes I think we are practicing it but at times you will find that other clients they go for long then they come here they have a long list of ailments that they report which at times gives us problems in giving that person fewer drugs, you find yourself, the client is so confused that they think asthmatic and also when they have flu at that time so you find now you are giving the long list (laughs)

**31:3 (pause) mmm we try to communicate with the rational pharmacist or some…… (2381:2497) - D 31: MA_zond**

(pause) mmm we try to communicate with the rational pharmacist or sometimes the national matron officer in the region

**31:4 yes we do have a regional pharmacist (laughs) but we don’t have a dire…… (2635:2731) - D 31: MA_zond**

yes we do have a regional pharmacist (laughs) but we don’t have a direct line with the pharmacist

**31:5 usually there is delay in delivering instead of sending the medicine;…… (3828:3965) - D 31: MA_zond**

usually there is delay in delivering instead of sending the medicine; we usually start ordering on the second week of the month every time

**31:6 and but sometimes we find they have problems that side then the orders…… (3999:4240) - D 31: MA_zond**

and but sometimes we find they have problems that side then the orders accumulate when we make another order, we order from what we have in the storeroom that time and you find out that they deliver that order times they deliver the old order

**31:7 at times it’s so bad yah the stock out sometimes they are very bad, yo…… (4314:4727) - D 31: MA_zond**

at times it’s so bad yah the stock out sometimes they are very bad, you even consider extend of negotiating with the health committee to help us, we sometimes buy from other pharmacies to help the client because at times there are major drugs that are out of stock and it’s difficult to work, we have difficult times even the instruction not to stock this medication, but its stock that we need how do you explain?

**31:8 I’m….. (Pause) if it’s possible for the ministry, if they can get a pe…… (5928:6055) - D 31: MA_zond**

I’m….. (Pause) if it’s possible for the ministry, if they can get a person trained for pharmacy I think that can help a lot, yes

**31:9 mhlawumbe with an update in training even the nurses can continue for…… (6086:6213) - D 31: MA_zond**

mhlawumbe with an update in training even the nurses can continue for a while, while they are working like an inservice training

**32:1 in my understanding, rational drug use is the use of specific drugs to…… (399:714) - D 32: SH_hlat....n malingaa**

in my understanding, rational drug use is the use of specific drugs to specific eeh ailments, aah that entails the number of drugs that we have used, that is taken by patient for that particular ailment. It has to be the method and the prescriber has to follow treatment guidelines correctly, yah I think that is it.

**32:2 There are prescribers who come with a long list and then when we have…… (902:1105) - D 32: SH_hlat....n malingaa**

There are prescribers who come with a long list and then when we have meetings that’s where we raise those points and then some of them they refrain from doing that and then they come to good practice.

**32:3 we have the PTC that is there, eeh that is the pharmacists’ therapeuti…… (1242:1650) - D 32: SH_hlat....n malingaa**

we have the PTC that is there, eeh that is the pharmacists’ therapeutic committee. It deals with eeh the essential drug use for the facility and eeh and it enforces the use of the guideline eeh what else?, it also deliberates on new drug or new intervention and then it helps the ministry to buy those new drugs for the facility so that they can be prescribed, they can be included in our essential drug list.

**32:4 I would say it has been functional until last year of 2016 that’s when…… (1737:2184) - D 32: SH_hlat....n malingaa**

I would say it has been functional until last year of 2016 that’s when we had our last meeting in 2016, 2017 it has been dormant for some time. We have some challenges, so far on eeh human resource, shortage of human resource that’s number one, secondly we don’t call the meetings, sometimes we try to call the meeting, only a few people appear then we abort the meeting, so we still have got a lot of challenge. I don’t know how we can change that

**32:5 sometimes yes mostly these days we are out of stock of most of our ant…… (3123:3339) - D 32: SH_hlat....n malingaa**

sometimes yes mostly these days we are out of stock of most of our antiepileptic drug, and psychotic drugs, yah and eeh previously we had eeh stock out on insulin yah sometimes we have stock outs sometimes they come.

**32:6 aah I can’t guarantee that one because you can’t go into your patients…… (4146:4388) - D 32: SH_hlat....n malingaa**

aah I can’t guarantee that one because you can’t go into your patients bedroom, and see what they are doing there but the information we give them at the window it’s enough for them to understand the importance of adhering to their medication.

**32:7 there is a bit of a challenge on that one because we don’t have a pres…… (4745:4860) - D 32: SH_hlat....n malingaa**

there is a bit of a challenge on that one because we don’t have a prescription record that remains in our department

**32:8 if a patient is prescribed those medications and then after the disper…… (4893:5142) - D 32: SH_hlat....n malingaa**

if a patient is prescribed those medications and then after the dispersing, we give the prescription to the patient, so if the patients comes back with the prescription it’s easy to see but if the patient has lost the prescription that’s a challenge.

**32:9 actually with the present one, they are not comfortable, I would say b…… (6769:6926) - D 32: SH_hlat....n malingaa**

actually with the present one, they are not comfortable, I would say because they hinted out that it’s for primary level we don’t have one for secondary level

**32:10 we need to actually introduce guidelines that are relevant to all the…… (7572:8170) - D 32: SH_hlat....n malingaa**

we need to actually introduce guidelines that are relevant to all the three levels that I would say and secondly we need fully functional laboratories for our sensitivity tests and all those things so that we can rationally use our antibiotics, cause right now we have a lot of antibiotics that are no more working I would say and the resistance at times is high you find that only one or two antibiotics are not resistant, and then the rest are resistant and when we give them to patients they won’t start to work.so if you have a laboratory you can test that and find which ones are not resistant.

**32:11 we need to actually introduce guidelines that are relevant to all the…… (7572:8169) - D 32: SH_hlat....n malingaa**

we need to actually introduce guidelines that are relevant to all the three levels that I would say and secondly we need fully functional laboratories for our sensitivity tests and all those things so that we can rationally use our antibiotics, cause right now we have a lot of antibiotics that are no more working I would say and the resistance at times is high you find that only one or two antibiotics are not resistant, and then the rest are resistant and when we give them to patients they won’t start to work.so if you have a laboratory you can test that and find which ones are not resistant

**32:12 yah this one is a big topic actually, it has to be taken up to our min…… (8289:8704) - D 32: SH_hlat....n malingaa**

yah this one is a big topic actually, it has to be taken up to our ministry, yah so that they can help us during close to our stock outs, more especially when you hire those medical officers from other regions, you find that they come their own practice from those regions which does not apply to our region here, so I do not know how they can maybe take them through before they post them to work in our facilities.

**32:13 thank you very much I have been thinking about that a lot we talk almo…… (10665:10968) - D 32: SH_hlat....n malingaa**

thank you very much I have been thinking about that a lot we talk almost every day about it, we have to do something about it. Some of the prescription we intend to even photocopy by unfortunately for Hlat we don’t have a photocopying machine, we don’t have paper, we don’t have anything to help us

**32:14 okay I was asking because in most cases in our country it is customary…… (9851:10039) - D 32: SH_hlat....n malingaa**

okay I was asking because in most cases in our country it is customary to just research thereafter you don’t get feedback, you don’t get any recommendation on what we are talking about so…

**33:1 I would say actually we are doing it but sometimes we are not doing it…… (833:1014) - D 33: SH_hlut**

I would say actually we are doing it but sometimes we are not doing it because there are not enough drugs or we are not used to the guideline especially understanding the guideline.

**33:2 I think the main challenge is not having a pharmacy assistant (2592:2652) - D 33: SH_hlut**

I think the main challenge is not having a pharmacy assistant

**33:3 cause we are not really sure on what we are supposed to do on that sid…… (2683:3048) - D 33: SH_hlut**

cause we are not really sure on what we are supposed to do on that side, on the pharmacy side so maybe we can order some drugs there is no one to tell us if we are doing the right thing or not, at the end of the day you won’t know when we are not receiving the drugs and when we receive some of them, we are not able to prescribe according to the standard guideline

**33:4 I am not sure what really the problem they are trying maybe but find t…… (3252:3559) - D 33: SH_hlut**

I am not sure what really the problem they are trying maybe but find that at the you first week of the month you find that maybe the order hasn’t got to the central medical stores because it’s a long route from the nurses to the sister then to the matrons’ office, from the matrons’ office back to Mbabane

**33:5 at the facility, first from the nurses, then to the sister, then the s…… (3624:3768) - D 33: SH_hlut**

at the facility, first from the nurses, then to the sister, then the sister sends it to the matron, from the matron to the central medical stores

**33:6 I think maybe it’s just that, sometimes there are no drugs at the cent…… (4285:4469) - D 33: SH_hlut**

I think maybe it’s just that, sometimes there are no drugs at the central medical stores because for instance nje we ordered from July, August, September but we only received in October

**33:7 pause) okay I think maybe we are partially doing it not because there…… (5943:6189) - D 33: SH_hlut**

pause) okay I think maybe we are partially doing it not because there is no stock of medicine. It could be just we don’t understand the essential and the guideline so maybe I think sometimes we need to sit down and have a look at it and analyse it

**33:8 I am not sure if I could add this one but if we could have some traini…… (6383:6500) - D 33: SH_hlut**

I am not sure if I could add this one but if we could have some training on issues of health, then we could do better

**33:9 even that one because well especially because we don’t have a pharmaci…… (6722:6946) - D 33: SH_hlut**

even that one because well especially because we don’t have a pharmacist or pharmtech cause its tricky, what are we supposed to do at the pharmacy how do I order, maybe we will understand better if someone is there to show us

**34:1 our challenges, some of the challenges yi-availability of the treatmen…… (1290:1733) - D 34: SH_lav**

our challenges, some of the challenges yi-availability of the treatment, accessibility to the clinic, it’s in town so everyone comes to the clinic even when there is no need just because they are passing by, they give you symptoms, things that are not even there sometimes so we end up prescribing treatment for people who don’t need it cause you can’t say they don’t need it angithi ma athi uyagula uyagula you “ cant say no you are not sick”

**34:2 why? Stock out maybe, mostly yi-stock out we order things and we won’t…… (2115:2328) - D 34: SH_lav**

why? Stock out maybe, mostly yi-stock out we order things and we won’t get them because they are out of stock and when we check the stock level esinaso sekuyophela sekude, and lama-expires, we have a lot of expires

**34:3 well sometimes we get a lot of drugs in bulk la ato-expire like soon,…… (2358:2474) - D 34: SH_lav**

well sometimes we get a lot of drugs in bulk la ato-expire like soon, soon, soon in a couple of months, yah that too.

**34:4 the order for last month they came on the last week of the month, the…… (3592:3962) - D 34: SH_lav**

the order for last month they came on the last week of the month, the following month yisikhatsi eyenta la kimi, ordering we do it monthly they keep to the office we don’t know how long it stays at the office because it goes to the central medical stores so we have such challenges and i-turnaround time you can’t even tell when we are having it ubona ngakho nje sekufile

**34:5 sometimes they come around to do the stock taking properly Kunapho esi…… (4541:4668) - D 34: SH_lav**

sometimes they come around to do the stock taking properly Kunapho esisitana khona with the monitoring of stuff and other things

**34:6 but there are buts obviously, yikuthi sengikhohliwe I had le-problem n…… (5768:6004) - D 34: SH_lav**

but there are buts obviously, yikuthi sengikhohliwe I had le-problem not even a long time back a couple of weeks back, sengikhohliwe ukuthi bengifuna ini but le kulencwadi bekukude i-dose yemntfwana, bekule adult dose kuphela on the book

**34:7 I think it was just that, there were no doses for children (6165:6222) - D 34: SH_lav**

I think it was just that, there were no doses for children

**35:1 yes exactly it’s that, I think they need to be revised, some of the th…… (2938:3046) - D 35: SH_mats**

yes exactly it’s that, I think they need to be revised, some of the things are very old, and they are history

**35:2 especially when prescribing antibiotics like in the guideline, there a…… (3646:3812) - D 35: SH_mats**

especially when prescribing antibiotics like in the guideline, there are a lot of cases whereby for each and every condition the antibiotic first level is amoxicillin

**35:3 and I think when it is overly prescribed it might lead to resistance d…… (3843:3966) - D 35: SH_mats**

and I think when it is overly prescribed it might lead to resistance down the way, we are not sure of our patients’ reaction

**35:4 because like it’s very effective, if someone is maybe complaining abou…… (3999:4260) - D 35: SH_mats**

because like it’s very effective, if someone is maybe complaining about upper resistance of the infection regardless if you give them CTM and amoxicillin and after two days you give them a different dose or continue with the same antibiotic, so we are not sure

**35:5 mmm it’s just that one more important thing is that in order to ration…… (4437:4978) - D 35: SH_mats**

mmm it’s just that one more important thing is that in order to rationally use drugs is that we have to have enough drugs. So there is a challenge now of the supplier like currently we don’t have proper medication also for hypertension, so that is a major challenge because you find that when the patient comes and there is no medication or we need to switch to another drug but we don’t have, or you find that this patient has been taking this particular drug, the following time the drug is also scarce so you have to switch to another one.

**35:6 yah stock out and not in the facility only but national level, it’s a…… (5072:5398) - D 35: SH_mats**

yah stock out and not in the facility only but national level, it’s a very big problem and things are bad. I think there in number of times when we placed orders and the central medical stores was out of stock and people keep on denying that they are not even there, they need to do something on that one, and it’s a challenge

**35:7 cause most of the time they make their appointments because they canno…… (7599:7963) - D 35: SH_mats**

cause most of the time they make their appointments because they cannot travel so I think maybe if someone is sent to a higher facility like this then it’s a big problem for them and us. I think they should be having a certain place for decentralising the problem, so like the nearest clinic and I think most clinics are capable of monitoring the particular patient

**36:1 so far we are practicing that but we have got a challenge with the sho…… (611:1257) - D 36: SH_mot**

so far we are practicing that but we have got a challenge with the short stuffing, we don’t have a pharmtech or someone who will just be assigned for the drugs or the storeroom only. I am saying so because so far we have got a lot of expired drugs really, really there is always no one is assigned to take care of the drugs in the store room. We are always busy with the clients; if you go to the storeroom and just unpacking fine when unpacking we do the C4 thing but when we start working there is no one who is answerable to the storeroom that is what is happening. When you go to the shops you find the drugs expired and they keep on expiring.

**36:2 it is somewhat big, but when you are used to using it you get used to…… (1840:2219) - D 36: SH_mot**

it is somewhat big, but when you are used to using it you get used to it, but it is user friendly but I would suggest if there would be pocket companion a small one that would be fine especially for someone new coming first from college or coming from the hospital if they come here we give them the guideline it’s very difficult for them to use.so I suggest a smaller pocket book

**36:3 but there is a slight change, we were over prescribing antibiotics (2672:2737) - D 36: SH_mot**

but there is a slight change, we were over prescribing antibiotics

**36:4 very much, very much, because some of us here we come from the clinic,…… (3179:3447) - D 36: SH_mot**

very much, very much, because some of us here we come from the clinic, we are just given the guideline, some of us we don’t have any experience so vele we need the training because most of the time we need our experiences now that I give someone this, this could happen

**36:5 yah it’s a pile of data. I would also suggest that if a pharmacist, ph…… (6210:6460) - D 36: SH_mot**

yah it’s a pile of data. I would also suggest that if a pharmacist, pharmtech or whatever be available like readily available in the facility maybe even come once in a week just to check our stock, our environment and everything should be fine for us.

**36:6 because thina we are working fine, but we don’t have that much time to…… (6491:7071) - D 36: SH_mot**

because thina we are working fine, but we don’t have that much time to just look at the pharmacy, to see what is there, what is not there, what is expiring and what is not expiring today. We really don’t have time, we just find out when we just want to use the medicine now it has expired or now it’s finished. Last time in November we ordered Amoxyl but the powdered one, but when we tried to dilute it, it was making some lumps. It wasn’t expired but the quality was bad they had to take it back, so there is no one to foresee that we just see it when we want to use it right now

**37:1 yah it is a question that we will be here for a long time if we try an…… (1384:2644) - D 37: SH_nho**

yah it is a question that we will be here for a long time if we try and understand. We don’t believe that there is rational drug use here. Ever since I started working here, I think almost seven years now but even my other colleagues will agree with me. We have been trying to intervene…, to do some guided interventions in terms of rational drug use, given the therapeutic committee for the hospital, but It’s not working cause if you find the prescription…, if we take the prescription for instance, we would see some other patient they gave a combination of different antibiotics, too many antibiotics or you can find someone given maybe thirteen drugs in one prescription despite the patient might be diabetic or hypertensive but you can still see the patient getting at least something like… getting more than five drugs at a time and you ask yourself is this patient treated well, is it going to help or is it going to cause other problem in future, but yes some things need to change so I don’t know. Sometimes we try contacting the prescribers but they still the same prescribers, they still prescribe the same thing the following day. It’s something that needs intervention for drug use maybe, I don’t know how they have to, but they need intervention

**37:2 right now it’s not functional, we had our meeting January this year, y…… (2773:4023) - D 37: SH_nho**

right now it’s not functional, we had our meeting January this year, yah I think so we had an issue about the meetings. Last year we had a problem that there were so many meetings and so many committees in the hospital and you find out that some of the committees they intervene in some of the issues that we talk about in the hospital including maybe the rational drug use like for example the inservice committee sometimes talk about the rational use of drugs and any topics that has to do with pharmacy, there is infection control, we have got quality control committee. Those committees they talk of maybe more like an incentive so there was a problem was that the turnout now of this meeting was low and even if you put the memos in time to say we have got a meeting maybe in a weeks’ time can you come, you find that a few people will come and it’s the issue of there are therapeutic committees, it’s like it’s not supposed to be a pharmacy issue, it’s supposed to be an office issue concerning maybe, it could be pharmacy and other department, but if they take it as maybe pharmacy, pharmacy baby for instance . So we had a challenge to try and convince them in the meetings, but however we need to maybe try again, to try and not to lose hope

**37:3 but the most problem issue about rational drug use is the use of antib…… (4485:4919) - D 37: SH_nho**

but the most problem issue about rational drug use is the use of antibiotics. The issue of antibiotics yah (laughs) is maybe… I don’t know what kind of intervention that we can do. Although sometimes for us as a pharmacy department we do talk to the prescriber if we see something like that, that is not okay in the prescription, we do consult them and we give them our opinion based on our materials that we have here in the pharmacy.

**37:4 so some of the prescribers, they won’t know how certain conditions are…… (4954:5892) - D 37: SH_nho**

so some of the prescribers, they won’t know how certain conditions are treated and we did last time I think Swaziland came up with the standard treatment guideline and each and every facility is supposed to be using that treatment book, but I am telling you right now, that book has never been used. We got those books from the central medical stores after some meetings… after some therapeutic committee meetings, after some inservice training we did give them, each and every department has got, if not all the nurses. We try and put those books in each and every consultation room but right now I think they are just gathering dust. They don’t even know, even if you ask them how this condition is treated according to research and the guideline, they don’t even know how to use them, us the pharmacy has to correct them, they don’t know which one is which, so there is more to do in terms of inservice training about certain conditions

**37:5 so some of the prescribers, they won’t know how certain conditions are…… (4954:5894) - D 37: SH_nho**

so some of the prescribers, they won’t know how certain conditions are treated and we did last time I think Swaziland came up with the standard treatment guideline and each and every facility is supposed to be using that treatment book, but I am telling you right now, that book has never been used. We got those books from the central medical stores after some meetings… after some therapeutic committee meetings, after some inservice training we did give them, each and every department has got, if not all the nurses. We try and put those books in each and every consultation room but right now I think they are just gathering dust. They don’t even know, even if you ask them how this condition is treated according to research and the guideline, they don’t even know how to use them, us the pharmacy has to correct them, they don’t know which one is which, so there is more to do in terms of inservice training about certain conditions.

**37:6 there is a…, one there is overdosing, and two there is underdosing esp…… (6087:6731) - D 37: SH_nho**

there is a…, one there is overdosing, and two there is underdosing especially in children, normally when we talk about children we talk about weight, we talk about age groups. So you find that some are getting a very high dose which does not follow the age group of that child, or they get an adult dose. So those are the problems. Well with the antibiotic issue, being an underdose it’s likely that you are going to carry resistance to that child, oh the other issue… is the issue of getting maybe like one condition and treating it with three different types of drugs that you are supposed to use but you start with the one that is in protocol

**37:7 yes the first line yes. You start with the first line. If you see the…… (6768:7715) - D 37: SH_nho**

yes the first line yes. You start with the first line. If you see the first line is not working like for example with amoxyl someone has a throat infection, you start with amoxyl. If you see that 125mlg is not working you then need to step up the dose and give let’s say maybe 250. If you see maybe that’s not working then maybe you can start changing the drug, change it to maybe dichromatic, domiciling or any other drug, but you cannot start by giving a child strong antibiotic or a very high dose. If that child comes again we give him amoxyl it’s likely that it won’t work for the child. So these are some of the issues that we see, last year a baby just came from… it’s a one day baby was prescribed kafanzol you see. So you are wondering ukuthi is this baby just starting cafonol, is she going to be able to do well with amoxyl if she is prescribed next time, you see we have those kinds of incidents, they show signs of irrational drug use.

**37:8 yes there was one when we did a… one of the meetings on therapeutic co…… (7828:8323) - D 37: SH_nho**

yes there was one when we did a… one of the meetings on therapeutic committee, there was a pharmaceutical message, you can sense that this meeting though is all about the rational drug use and she came and gave a lecture about rational drug use. She came I think twice now, first she was under ministry then under MSH, she came and she did also a lecture on…, she talked about rational drug use, and we thought maybe people will understand, because that was like an inservice training but still…

**37:9 aah I would like to say if the ministry or the authority could try and…… (8507:8986) - D 37: SH_nho**

aah I would like to say if the ministry or the authority could try and help us in terms of more training, in terms of antibiotic, maybe in terms of all the drugs so that when we go to workshops lets’ say it’s about diabetics’ we go to be trained about those kinds of conditions and know how they are treated so that when the prescribers come back to their work station, they will know the proper way to treat that condition, not to write any how the way they think from their head

**37:10 because now another challenge that we have is the issue of chronic pat…… (9027:9807) - D 37: SH_nho**

because now another challenge that we have is the issue of chronic patients especially hypertensive patients those ones who are suffering from diabetics, you find that some of the drugs that they get, some of them are not necessary especially to do with anti-hypertension, the patient cannot just come here initially for it to be diagnosed with eeh… be it hypertension, and you start there and there to give maybe three drugs of antihypertension. Maybe it will start with HCTZ, HCT you see how it goes, if you see then that it’s not controllable then you add another drug, then another drug, you don’t just start with three to four drugs on one patient so those kind of things are really what they are doing so we really need an intervention, we need more workshops, more training

**37:11 this issue of the therapeutic committees though we don’t want them to…… (9842:10162) - D 37: SH_nho**

this issue of the therapeutic committees though we don’t want them to think that it’s a pharmacy issue only when it’s an issue that involve all the departments, yes so when we say we are going to have a meeting we are all going to with one mind not to think it’s the pharmacy who is going to tell us what they want to do

**38:1 as a facility I would say we are but sometimes it’s challenging becaus…… (650:1160) - D 38: SH_nhlangj**

as a facility I would say we are but sometimes it’s challenging because the patient at times comes with a condition then you will think twice whether it is you , are you giving the right medication on the condition and then it’s hard for you to consult the guideline in front of the patient so yah but nje we usually do some meetings maybe and then we consult with the guideline, we pick some conditions and then we study them and see how we could administer particular medication for that particular condition

**38:2 in our facility it is not the nurse, it is the orderlies (2705:2760) - D 38: SH_nhlangj**

in our facility it is not the nurse, it is the orderlies

**38:3 but then I always tell my patients, if the guy or the lady who is goin…… (3041:3280) - D 38: SH_nhlangj**

but then I always tell my patients, if the guy or the lady who is going to give you the medication and when you asking ‘what is this medication for’, and then they don’t have the answer for you please come back so that I can explain to you.

**38:4 our orders are always on time but maybe we do have… we are also to bla…… (5088:5739) - D 38: SH_nhlangj**

our orders are always on time but maybe we do have… we are also to blame maybe because I have learnt, we have learnt that in the last month we used to order one month stock yet we are supposed to order for three months so maybe that too has something to do with us running out of stock, but again I think also central medical stores have to take a blame at some point because we always order but then they are not giving us back what we ordered and there is no communication whatsoever so the we get confused. We don’t know what to do we complain they just tell us that they are cutting the stock, it’s been there at the end of the day there is nothing

**39:1 we have. We do have a copy of the guideline also at the consultation r…… (2002:2246) - D 39: SH_Nkwen**

we have. We do have a copy of the guideline also at the consultation room, we do have it it’s just they are misplaced because we were doing renovation some of things are just packed we just packed, we just go and unpack they if we want to use it

**39:2 When we have a challenge they pave the way sometimes and when they are…… (2417:2548) - D 39: SH_Nkwen**

When we have a challenge they pave the way sometimes and when they are lying and people are shouting at times we don’t go via them.

**39:3 we do need not to have the stock out to take too long it should be a s…… (4777:5086) - D 39: SH_Nkwen**

we do need not to have the stock out to take too long it should be a short period of time cause we are looking at the community most of the people don’t have money to go and buy the medicine they should have, If it should be a period of one week it must be a week not a period of six months we don’t have any.
