# Supplementary material for: Health system actors’ perspectives of prescribing practices in public health facilities in Eswatini: A Qualitative Study
Source: PLoS One. 2020 Jul 9;15(7):e0235513. doi: 10.1371/journal.pone.0235513 (PMC7347100; doi:10.1371/journal.pone.0235513)
Supplement: Attachment — Submitted filename: Response to reviewers comments.docx [file pone.0235513.s011.docx]

School of Public Health

University of the Western Cape

Private Bag X17

Bellville 7535

South Africa

1 June 2020

The Editor in Chief

PLos One Journal

Dear Sir/Madam

Re: **RESPONSE TO PLOS ONE REVIEWERS’ COMMENTS**

The researchers wish to thank the editor and reviewers for the time availed to review the manuscript and the valuable comments offered. Below are itemized replies to the comments. The reviewers’ comments are reproduced for ease of reference, followed by the response of the researchers. Modifications are in track changes in the manuscript entitled
“Revised Manuscript with Track Changes”.

Anonymised data sets have also been uploaded.

Editor’s Comments

**Comment:** Add one key reference on qualitative research reporting guidelines.

**Response:** Qualitative research reporting guidelines have been referenced in the manuscript on page 25, 1^st^ paragraph.

**Comment:** Check and revise throughout in line with COREQ 32 item checklist for qualitative studies.

**Response:** The manuscript has been revised in line with the COREQ 32 item checklist and the checklist has been uploaded as a supporting file.

**Comment:** Reorganize the sequence of Results section in line with key themes emanated [LINES: 188-190]

**Response:** The Results section has been reorganized in line with key themes – material, meaning, and competence – on pages 12-22.

Journal Requirements

**Comment:** Please ensure that your manuscript meets PLOS ONE's style requirements, including those for file naming.

**Response:** The manuscript has been revised to meet PLOS ONE's style requirements, including those for file naming.

**Comment:** Please ensure you have thoroughly discussed any potential limitations of this study within the Discussion section.

**Response:** Study limitations have been discussed on page 25 of the manuscript under the section “Study limitations”.

**Comment:** Please include additional information regarding the interview guide used in the study and ensure that you have provided sufficient details that others could replicate the analyses.

**Response:** Detail on the interview guide has been added under the “Data collection” section on pages 10-11, and under the section “Trustworthiness and rigour” on page 24; paragraph 1, lines 4-6. The interview guides (for KIs and facility managers) have also been uploaded as supporting files.

**Comment:** Please provide additional details regarding participant consent. In the ethics statement in the Methods and online submission information, please ensure that you have specified what type of consent you obtained (for instance, written or verbal, and if verbal, how it was documented and witnessed).

**Response:** Additional detail on participant consent has been added under the section “Ethical consideration” on page 11; lines 6-9.

**Comment:** We note that you have indicated that data from this study are available upon request. PLOS only allows data to be available upon request if there are legal or ethical restrictions on sharing data publicly.

**Response:** This has been rectified on the submission. For this study there are no legal/ethical restrictions on publicly sharing the data.

Comment: PLOS requires an ORCID iD for the corresponding author in Editorial Manager on papers submitted after December 6th, 2016. Please ensure that you have an ORCID iD and that it is validated in Editorial Manager.

**Response:** The corresponding author now has an ORCID iD.

Reviewer 1 Comments

**Introduction**
**Comment:** It is not clear how the Social Practice Theory developed in this section informed the method section. It seems as you used elements of the SPT to develop your interview guide.
I would rather suggest that the authors develop a sub-section on “Conceptual framework” under the methods section.

**Response:** The authors used the SPT elements to develop the interview guides for KIs and facility managers – this has been clarified under the “Conceptual Framework” section on page 8.

**Methods**
**Comment 1:** Study design: This should provide a clear statement on the study design, with appropriate definition and referencing. The authors state that this is an ‘qualitative exploratory study design’. This will need defining and referencing.

**Response 1:** This has been addressed under the “Study design and sampling” section on page 8; paragraph 1; lines 1-4.

**Comment 2:** The authors should describe how this study is embedded in the larger intervention study, in term of research questions, methodology, ….. How are they complementary?

**Response 2:** This has been explained under the “Study design and sampling” section on pages 8-9.

**Comment 3:** The sampling requires a clear summary about the people who were interviewed, especially frontline managers. How they were sampled, and how they were recruited. In addition, how the balance between urban/rural settings, public/private health facilities was ensured.

**Response 3:** random sampling was conducted for the big study as detailed under the “Study design and sampling” section. Frontline managers came from the randomly sampled facilities. Sampling detail has been added on pages 8-10.

I would propose that the following information is covered:
**Comment 4:** A sentence stating how these were sampled, including a definition of this sampling strategy, an explanation about why this sampling strategy was used, and a recognisable reference to support these ideas.

**Response 4:** This has been done under the “Study design and sampling” section on pages 8-10.

**Comment 5:** A table to clearly outline and summarize the sample, including information on number of participants, district participants, settings, level of care, owner of the facility (public/private).

**Response 5:** This has been clarified in the manuscript. Selection of included facilities is explained under the “Study design and sampling” section on page 8; paragraph 2; lines 5-14. Participant characteristics are further unpacked in Table 2 on pages 9-10.

**Comment 6:** A very transparent explanation about how these participants were recruited into the study (how were they identified, how were they invited into the study, who invited them into the study etc).

**Response 6:** This has been clarified on page 11, paragraph 2; lines 1-4.

**Comment 7:** A statement on whether they were reimbursed/incentivised.

**Response 7:** This has been done under the “Ethical considerations” section on page 11; paragraph 1, lines 6-7.

**Data collection**
The data collection subsection should include a clear account of the following issues:
**Comment 1:** Why a semi-structured interview was chosen, with a reference to literature

**Response 1:** This has been addressed under the “Data collection” section on page 10; paragraph 1, lines 8-10.

**Comment 2:** How long interviews lasted

**Response 2:** This has been addressed under the “Data collection” section on page 10; paragraph 1, lines 12-13.

**Comment 3:** What language they were conducted in

**Response 3:** This has been addressed under the “Data collection” section on page 10; paragraph 1, lines 12-13.

**Data Analysis**
The analysis process needs to be clearly described, and referenced to a recognisable analysis process. This section lacks convincing or transparent explanation. Please clarify so there are clear statements on:
**Comment1:** The analysis process used, with reference to a systematic analysis process

**Response 1:** This has been addressed under the “Data analyses” section on page 12; paragraph 1.

**Comment 2:** a step by step explanation about how the analysis was conducted, including an explanation of which analysis approach and technique (again with references) were used in this study.

**Response 2:** This has been addressed under the “Data analyses” section on page 12; paragraph 2.

**Results**
**Comment 1:** Lines 186-188 should do under methods section.

**Response 1:** Lines 186-188 have been moved to the “Methods” section under “Study design and sampling”

**Comment 2:** The reference for each quote needs to be done in a systematic fashion, to provide sufficient information linking the quote back to the sample. I propose that the authors include ‘role, level of work, district interviewee is from’. These need to link back clearly to the sample section of the methods, and to the table summarizing the sample, so readers are clear who is quoted. At the moment, we have no idea how many of the interviewees are represented in the quotes selected, and how representative of the sample these selected quotes are.

**Response 2:** This has been addressed for all quotes. Each quote is referenced by Key Informant/Facility Manager_ Cadre_Level of Care_Region

**Discussion**
The authors should discuss the study limitation: methods and results

**Comment 1:** The limitation is applying the Social Practice Theory

**Response 1:** This has been addressed under the “Study limitations” section on page 25.

**Comment 2:** The limitation in utilising the adapted model of practice (SPT)

**Response 2:** The authors revised the wording in the manuscript and used the SPT and not an adapted model of SPT. Limitations of using the SPT are covered under the “Study limitations” section on page 25.

**Comment 3:** The study limitations section needs to include statements on the ‘reliability’ and ‘rigour’ of the study design. Explanations of these are given in any good methods text book (e.g. Bryman, A. (2008). Social Research Methods. Oxford: Oxford University Press)

**Response 3:** This has been addressed under the “Study limitations” section on page 25.

**Comment 4:** In the study limitations, you will also need to account for the potential issues associated with rigour and integrity associated with using a number of different topic guides throughout the study, as it ‘thins’ the data collected (i.e. not all interviews were conducted in the same manner)

**Response 4:** This has been addressed under the “Trustworthiness and rigour” section on page 24; paragraph 1.

Reviewer 2 Comments

**Introduction**
**Comment 1:** The authors need to provide more context on the Standard Treatment Guidelines (STGs) and the Essential Medicines List (EML). Are the medicines in these documents assigned specific levels of care? Are there key differences between the EML and STGs? For example, do these documents differ on how they assign/restrict medicines to levels of care? These are needed to put the findings from the study in context.

**Response 1:** This has been addressed under the “Introduction” section on pages 5-6.

**Methods**
**Comment 1:** Under study setting, it would be helpful to also indicate which cadres of health workers are available at the various levels of care.

**Response 1:** This has been addressed under the “Study setting” section on page 7; paragraph 1, lines 9-15.

**Results**
**Comment 1:** The authors provided the range of the number of years of experience of the study respondents. It would be helpful to also provide the median number of years of experience.

**Response1:** The median number of years of experience has been added under the “Results” section on page 12; paragraph 1; line 3.

**Comment 2:** It would be helpful to include the breakdown of the number of respondents (especially the 32 frontline health workers) by level of care or type of facility (clinics, health centres, hospitals etc.)

**Response 2:** This has been addressed in Table 2 under the “Study design and sampling” on pages 9-10.

**Comment 3:** It is hard to believe the STG/EML were mainly tailored for primary health care as reported by the authors: “current STG/EML, which were mainly tailored for primary health care” (under the section titled “availability and use of STG/EML”). This may require further explanation. Does this mean the medicines in the EML for instance, are only medicines for primary care services? The comment that “Frontline healthcare providers highlighted the difficulty for them to adhere to treatment guidelines due to restrictions on availability of medicines at certain levels of care” (under the section titled “restriction of medicines by level of care”) indicates that the guidelines may not only tailored to primary care facilities

**Response 3:** The wording has been changed from “tailored to “targeted at” on page 13 under the section “Availability and use of the STG/EML” and we have further explained why the guidelines were targeted at primary health care in lines 3-8.

**Comment 4:** This finding/sentence may also need more clarification: “However, key informants highlighted that targeting the guidelines at primary level of care resulted in secondary and tertiary level facilities prescribing outside the guidelines”. What does “targeting guidelines” mean? Does it have to do with the content of the guidelines focusing on primary care or the promotion of the guidelines to primary care facilities?

**Response 4:** The wording in the section “Availability and use of the STG/EML” line 9 has been changed from “targeting the guideline” to “targeting the STG” as the STG is targeted at primary healthcare while the EML covers all levels of care.

**Comment 5:** Line 268 -269: Please provide specific examples of NCD medicines and antibiotics that are not available at primary health care facilities.

**Response 5:** Specific examples of NCD medicines and antibiotics not available at primary level of care have been provided under the section “Restrictions of medicines by level of care” on page 15; paragraph 2; lines 3-14.

Yours Faithfully,

Nondumiso BQ Ncube
