# Supplementary material for: Health system actors’ perspectives of prescribing practices in public health facilities in Eswatini: A Qualitative Study
Source: PLoS One. 2020 Jul 9;15(7):e0235513. doi: 10.1371/journal.pone.0235513 (PMC7347100; doi:10.1371/journal.pone.0235513)
Supplement: Attachment — Submitted filename: Response to reviewers comments.docx [file pone.0235513.s012.docx]

School of Public Health

University of the Western Cape

Private Bag X17

Bellville 7535

South Africa

17 June 2020

The Editor in Chief

PLos One Journal

Dear Sir/Madam

Re: **RESPONSE TO PLOS ONE REVIEWERS’ COMMENTS**

The researchers wish to thank the editor and reviewers for the time availed to review the manuscript and the valuable comments offered. Below are itemized replies to the comments. The reviewers’ comments are reproduced for ease of reference, followed by the response of the researchers. Modifications are in track changes in the manuscript entitled
“Revised Manuscript with Track Changes”.

Anonymised data sets have also been uploaded.

The authors do not wish to make any modifications to the financial statement.

Reviewer 1’s Comments

**Introduction**

**Comment:** The statement “In 2017, posts for pharmacy assistants in all levels of care, but mainly clinics, were approved” is not clear. I suggest the authors expand on this statement.

**Response:** This comment has been addressed and the sentence improved on page 4; lines 84-86 in the manuscript.

**Methods**

**Comment:** I find it confusing that the “theory informed implementation intervention (the theoretical domains framework)” was introduced in the methods section (under the section titled, conceptual framework), even though this is not the theoretical framework used for this qualitative study. I agree it will be nice to have a subsection on the conceptual framework in the methods section. However, I believe this subsection should focus on the framework that is relevant for the methodology of this particular study. This will be a good place to discuss how the framework informed the design of interview guides etc.

**Response:**  The information and graphic on the theoretical domains framework has been deleted and the social practice theory has been used as it is relevant for this study. Information on how the SPT was used to design interview guides is available on page 7; lines 153-156.

**Data Management**

**Comment:** I do not believe it is necessary to disclose what the codenames of the transcripts mean. A general statement that the data were anonymized, and stored securely should suffice.

**Response:** The information has been modified on page 9; line 191.

**Data analysis**

**Comment:** It seems the information in the second paragraph should come before the first paragraph.

**Response:** The paragraphs have been re-arranged on page 9; paragraphs 1 and 2.

**Study design and sampling**

**Comment:** I do not believe it is necessary to talk about the sampling of prescriptions which was done in the larger study but irrelevant (and perhaps distracting) for this qualitative study. Since the qualitative interviews were with health facilities, the description on how these facilities were sampled should be enough.

**Response:** The information on sampling of prescriptions has been deleted and only information on sampling of facilities left in the manuscript on page 5; lines 115-117.

**Results**

**Comment 1:** Availability and use of STG/EML: The authors stated, “Information from key informants highlighted that public sector prescribing practices were influenced by the current STG/EML, which were mainly targeted at primary health care (though they can still be used at secondary and tertiary levels of healthcare)”.. the authors should revise the first sentence of this section which says that both the STG and the EML are focused on primary care.

**Response 1:** The sentence has been modified on page 10; lines 232-235.

**Comment 2:** Restriction of medicines by level of care: The authors stated, “Frontline healthcare providers highlighted the difficulty for them to adhere to treatment guidelines due to restrictions on availability of medicines at certain levels of care. An example cited was the unavailability of ceftriaxone and azithromycin at primary healthcare levels yet the latest guidelines for managing sexually transmitted infections recommend these medicines as first line therapy and for them to be available at primary healthcare level.” This is one example where referring to the STGs and the EML as if they were one document could mask important findings that may have implications for the revision of these documents. As I stated in the introduction, the EML restricts medicines to specific levels of care while the STG does not. This should be made clear in the presentation of the findings in the above sentence. If I understand correctly, based on the EML, primary care level facilities are not allowed to stock certain medicines. However, the STGs expect these facilities to treat conditions that require the use of medicines the EML restricts to higher level facilities. So there is a discordance between the EML and the STGs which may be impacting rational use of medicines.

**Response 2:** Clarifications on information from respondents around the STG and EML have been made on page 10; lines 232-242.

Yours Faithfully,

Nondumiso BQ Ncube
